# Fingerprinting hyperglycemia using predictive modelling approach based on low-cost routine CBC and CRP diagnostics

**DOI:** 10.1038/s41598-023-44623-4

**Published:** 2024-01-11

**Authors:** Amna Tahir, Kashif Asghar, Waqas Shafiq, Hijab Batool, Dilawar Khan, Omar Chughtai, Safee Ullah Chaudhary

**Affiliations:** 1 Biomedical Informatics and Engineering Research Laboratory, Department of Life Sciences, Syed Babar Ali School of Science and Engineering, Lahore University of Management Sciences, Lahore, Pakistan; 2 Basic Science Department, Shaukat Khanum Memorial Cancer Hospital and Research Centre, Lahore, Pakistan; 3 Department of Internal Medicine, Shaukat Khanum Memorial Cancer Hospital and Research Centre, Lahore, Pakistan; 4 Chughtai Institute of Pathology, Lahore, Pakistan

**Keywords:** Predictive markers, Prognostic markers, Endocrinology, Biomarkers

## Abstract

Hyperglycemia is an outcome of dysregulated glucose homeostasis in the human body and may induce chronic elevation of blood glucose levels. Lifestyle factors such as overnutrition, physical inactivity, and psychosocials coupled with systemic low-grade inflammation have a strong negative impact on glucose homeostasis, in particular, insulin sensitivity. Together, these factors contribute to the pathophysiology of diabetes (DM) and expanding landscape of its prevalence regionally and globally. The rapid rise in the prevalence of type 2 diabetes, therefore, underscores the need for its early diagnosis and treatment. In this work, we have evaluated the discriminatory capacity of different diagnostic markers including inflammatory biomolecules and RBC (Red Blood Cell) indices in predicting the risk of hyperglycemia and borderline hyperglycemia. For that, 208,137 clinical diagnostic entries obtained over five years from Chugtai Labs, Pakistan, were retrospectively evaluated. The dataset included HbA1c (n = 142,011), complete blood count (CBC, n = 84,263), fasting blood glucose (FBG, n = 35,363), and C-reactive protein (CRP, n = 9035) tests. Our results provide four glycemic predictive models for two cohorts HbA1c and FBG) each having an overall predictive accuracy of more than 80% (p-value < 0.0001). Next, multivariate analysis (MANOVA) followed by univariate analysis (ANOVA) was employed to identify predictors with significant discriminatory capacity for different levels of glycemia. We show that the interplay between inflammation, hyperglycemic-induced derangements in RBC indices, and altered glucose homeostasis could be employed for prognosticating hyperglycemic outcomes. Our results then conclude a glycemic predictor with high sensitivity and specificity, employing inflammatory markers coupled with RBC indices, to predict glycemic outcomes (ROC p-value < 0.0001). Taken together, this study outlines a predictor of glycemic outcomes which could assist as a prophylactic intervention in predicting the early onset of hyperglycemia and borderline hyperglycemia.

## Introduction

Hyperglycemia is an outcome of altered glucose homeostasis due to impaired insulin secretion and varying degrees of peripheral insulin resistance^1^. Hyperglycemia is a key component in the pathophysiology of diabetes due to glucose dysregulation^2^. Multiple lifestyle factors like rapid urbanization, aging populations, and increasing obesity due to sedentary lifestyle, inflammation, comorbidities, and genetic risk factors have put an ever-increasing number of people at risk of developing diabetes mellitus (DM)^2,3^. An epidemiological study on the global prevalence of diabetes reported that the prevalence of diabetes for all age groups will reach 4.4% of the human population in 2030^3^. Further, The International Diabetes Federation (IDF) reports that approximately 537 million people in the world suffer from diabetes^4^, out of which around 88,000 patients end up losing their lives every day^5^.

In the specific case of Pakistan, world’s 5th populous country^6^, studies estimating prevalence of diabetes are showing an alarming increase in prevalence of the condition across all segments of the population^7–9^. Limited governmental support for monitoring and controlling DM is extracting a hitherto unaccounted cost from the public in the form of poor quality of life for those suffering from diabetes. In 2017, Pakistan had 7.4 million cases of diabetes which accounted for 6.9% of the total adult population^4^. In 2022, this rate has rapidly increased to 26.7% with a total of 32.9 million diagnosed cases^4^. It is important to note that unmonitored diabetes can give rise to micro- and macrovascular diseases including 37% of cases of kidney failure^10^, 40% of cardiovascular events, 14% of heart attacks, and 10% of strokes^11^. Furthermore, according to “*Diabetes statistics in Pakistan*”, women exhibit a higher prevalence of diabetes than men^5^, thereby increasing their susceptibility to heart diseases, stroke, renal failure, or other life-threatening infections manifesting as complications attributed to hyperglycemia. In particular, it has been reported that women with diabetes are 23% more prone to heart disease in comparison to those without diabetes^5^.

In terms of diagnostics, HbA1c (glycated hemoglobin) is an established bio-indicator in providing the cumulative glycemic history of an individual of the preceding two to three months ^12^. American Diabetes Association (ADA) has recommended HbA1c as key diabetes marker^13,14^, besides its additive use as an independent risk factor for evaluating heart diseases and stroke in cases with or without diabetes^12^. This highlights its importance as a biomarker that is capable of providing information not only for the diagnosis and prognosis of DM but also for adverse outcomes resulting from it. In addition to raised levels of HbA1c, poor lifestyle choices also lead to insulin resistance via local and systemic low-grade inflammation ^15–17^. This further expands the role of inflammation in the pathophysiology of type-2 diabetes and associated metabolic disorders. Consequently, it is imperative to additively focus on inflammation for timely prevention and better control of diabetes^18^. Hematologic diagnostics such as complete blood count (CBC) have the potential to provide meaningful insights into hyperglycemia-induced irregularities in RBC (Red blood cells), WBC (white blood cells), and PLT (Platelets). Onwards analysis of these parameters in concomitance with inflammation can provide a valuable tool for studying the progression and management of diabetes besides evaluating the development of the degenerative micro and macro-vascular complication in DM^19^.

Higher levels of WBCs and their subtypes (neutrophils, monocytes, lymphocytes, and eosinophils), platelet count, and inflammatory markers like CRP (C-reactive protein) are associated with an increased risk of cardiovascular diseases and diabetes ^20–22^. Both WBCs and CRP are non-specific markers for systemic inflammation but carry comparable power for predicting heart diseases ^23–26^. Moreover, NLR (neutrophil to lymphocyte ratio) and PLR (platelet to lymphocyte ratio) are established systemic inflammatory response indices and are associated with diabetes-associated complications and hence useful in predicting DM^27–29^. Additionally, Erythrocytes are the most abundant cells in the blood and very sensitive to the changes in the composition of plasma ^19,24,30^. Consequently, due to long-term hyperglycemia, morphology, metabolism, and functions of the RBCs are subjugated to inevitable changes, which further affects hemorheology and microcirculation^31^. Taken together, these parameters which are indicative of RBC’s morphology, function, and production can be further employed to assess diagnosis and risk analysis of other diseases including diabetes^19,30,32^. Summarily, the overall temporal burden of hyperglycemia accounts for diabetes and related complications toward poor health outcomes ^33,34^. Moreover, predicting glycemic outcomes using simple diagnostic laboratory tests remains limited ^19,30,35^.

In this study, we aim to integratively investigate the interplay between inflammation, RBC parameters, and hyperglycemia by employing clinical diagnostics of CBC, CRP, HbA1c, and FBG (fasting blood glucose) tests towards developing a predictive model of glycemic outcomes. The study then envisages employing inflammatory (WBC, NLR, PLR, and CRP) and molecular (RBC, MCV, and MCH) markers to undertake risk profiling in normal, borderline, and hyperglycemic states. The resultant glycemic predictor will provide a discriminatory function encompassing the aforementioned markers, toward estimating the probability of an individual’s glycemic outcomes. Consequently, the study will provide an economical and efficacious risk fingerprinting tool for use with the routine screening of individuals with borderline hyperglycemia (HbA1c: 5.7%- 6.4%) for prognosticating the onset of diabetes as well as its control and management.

## Methods

### Data collection of clinical measurements

Pseudonymized clinical diagnostics data for 5 years (January 01, 2017, to June 08, 2022) was acquired from Chugtai Lahore Laboratory. The dataset included spatially distributed samples across 70 different cities in Pakistan. The study design was approved by the institutional review boards (IRB) of both the affiliated institutions. Where, the individuals and/legal guardian(s) walking in for diagnostic testing provided full informed consent of usage of their data for any future research carried under Chugtai Labs. Further, all the procedures performed in this study were in accordance with the ethical standards of the institutional research committee. After data filtration and cleaning, a total sample size of 208,137 diagnostic entries was concluded for onward analysis. The sample consisted of 79,221 females and 75,997 males aged from 10 to 85 years. The diagnostic measurements accounted for a total of HbA1c (n = 142,011), complete blood count (CBC, n = 84,263), fasting blood glucose (FBG, n = 35,363), and C-reactive protein (CRP, n = 9035) tests. For predictive modeling in association with different levels of glycemia, sub-cohorts were derived in the following combinations, HbA1c + CRP (n = 3,750), HbA1c + CBC (n = 50,124), FBG + CRP (n = 776) and FBG + CBC (n = 13,865) of tests.

### Study population and selection criteria

HbA1c and FBG—the two most common glycemic indicators, were utilized as grouping variables for splitting the sample into two sub-cohorts for the classification of glycemic status. The criteria defined by the American Diabetes Association (ADA) in “Classification and Diagnosis of Diabetes: standards of medical care in diabetes—2022” ^14^ were used to classify three levels of glycemia. An individual was classified to have (1) *hyperglycemia*, if they fulfilled any one of the following criteria: Fasting blood glucose FBG > 126 mg/dl and HbA1c > 6.5%, (2) *borderline hyperglycemia,* if 100 < FBG < 125 mg/dl and 5.7 < HbA1c < 6.4%, and (3) *normoglycemia,* if FBG < 100 mg/dl and HbA1c < 5.7%.

Henceforth, two separate sub-cohorts were then defined as (i) HbA1c cohort where the Individual’s glycemic status (hyperglycemia, borderline hyperglycemia, and normoglycemia) was categorized under ADA-defined HbA1c thresholds, ii) FBG cohort where individuals were grouped by ADA defined FBG thresholds.

### Statistical analysis

International Business Machine (IBM)—Statistical Package for Social Sciences (SPSS) version 26^36^ and R software program (version 4.2.2; R Foundation for Statistical Computing)^37^. Using the values from the neutrophils, lymphocytes, and platelets provided in the CBC test, NLR (Neutrophil to lymphocyte ratio) and PLR (Platelets to lymphocyte ratio) were calculated. Means and standard deviations for all the inflammatory markers (WBC, NLR, PLR, CRP, and Platelets) and RBC parameters (RBC, HCT, Hb, MCV, MCH, and MCHC) were calculated for two cohorts. Multivariate Analysis of Variance (MANOVAs) was calculated separately for two cohorts; first checking for the dependency of inflammatory markers on three stages of glycemia and later investigating the effect of glycemia on RBC status superimposed on inflammation. We found the p-value to be identical (< 0.0001) for Hotelling’s Trace, Pillai's Trace, Roy's Largest Root, and Wilks' Lambda statistics for all the sub-cohorts tested. Pillai’s trace was reported as the most robust and powerful statistic of any violations of assumptions. The significant MANOVAs were followed by individual one-way ANOVAs which were further followed by the LSD method for multiple comparisons analysis adjusting for α = 0.05. ANOVAs are robust to tests of homogeneity and, in general, Levene’s test was used to evaluate the equality of variance for each univariate test as it is less sensitive to departure from normality.

### Linear discriminant analysis (LDA) for model development

Significant ANOVAs (with p-value < 0.05) that showed more relevance to the three levels of glycemia were filtered and these as predictors were then used to develop a model with high predictive accuracy. LDA classifier’s results which provide the best linear combination of predictors to optimize the separation between multiple classes were used to develop Four LDA models, two for each cohort. The first two models represent the influence of only inflammation in predicting the glycemic status while the remaining two utilized the discriminatory power of both the inflammatory and molecular markers in predicting the glycemic outcomes.

### Model evaluation and ROC analysis

Two validation techniques were applied to assess the accuracy and robustness of the established models (i) the Back-substitution method by comparing the predicted classification of the discriminant function and the actual classification, the correct discriminant proportion of the classification function was calculated, and (ii) Jackknife (Leave-One-Out cross-validation). In model evaluation ROC analysis was performed, where a ROC curve shows the trade-off between the true positive fraction (TPF) and a false positive fraction (FPF) hence, providing a measure of sensitivity, specificity, and validity of our glycemic state predictor. The area under the curve (AUC) was measured with a 95% confidence interval and significance value.

## Results

### Inflammatory markers and RBC status exhibit concomitance with glycemic variations

Data on clinical diagnostics with a total sample size (n = 208,137) was obtained. Two sub-cohorts were derived according to HbA1c (n = 142,011) and fasting blood glucose (FBG, n = 35,362) tests. Means (± SD) for 12 different clinical parameters were measured for the glycemic states categorized under HbA1c (Table 1A) and FBG (Table 1B).

**Table 1:** Clinical diagnostic characteristics stratified by glycemic states using American Diabetes Association (ADA) thresholds.

(A) Glycemic levels categorized under HbA1c grouping							
Parameters		Normoglycemia		Borderline hyperglycemia		Hyperglycemia	
		HbA1c < 5.7% (n = 25,355)		5.7% < HbA1c < 6.4% (n = 30,072)		HbA1c > 6.4% (n = 86,584)	
Age (years)	Female	12,123 (47.81%)	45 ± 15	13,581 (45.16%)	55 ± 12	40,285 (46.52%)	55 ± 12
	Male	13,227 (52.18%)	48 ± 14	16,484 (54.81%)	54 ± 14	46,286 (53.45%)	54 ± 13
HbA1C (%)		5.3 ± 0.3		6.1 ± 0.2		8.3 ± 1.6	
CRP (mg/dL)		1.29 ± 3.1		1.73 ± 3.75		2.37 ± 4.26	
WBC (× 10^9^/L)		8.35 ± 4.4		8.41 ± 2.47		8.91 ± 3.4	
Platelets (× 10^9^/L)		266 ± 82		267 ± 86		271 ± 88	
NLR		2.22 ± 1.62		2.14 ± 1.47		2.22 ± 1.77	
PLR		9.59 ± 7.18		9.4 ± 6.26		9.75 ± 7.26	
RBC (× 10^12^/L)		4.81 ± 0.7		4.86 ± 0.67		4.92 ± 0.68	
MCH (pg)		27.8 ± 3.1		27.5 ± 3.1		27.1 ± 2.9	
MCHC (g/dL)		32.7 ± 1.5		32.5 ± 1.5		32.5 ± 1.5	
MCV (fl)		84.8 ± 7.8		84.3 ± 7.7		83.0 ± 7.3	
HCT (%)		40.6 ± 5.9		40.8 ± 5.5		40.8 ± 5.5	
Hb (g/dL)		13.3 ± 2.1		13.3 ± 1.9		13.2 ± 2.0	

(B) Glycemic levels categorized under fasting blood glucose (FBG) grouping							
Parameters		Normoglycemia		Borderline hyperglycemia		Hyperglycemia	
		FBG < 100 mg/dL (n = 11,061)		100 mg/dL < FBG < 125 mg/dL (n = 10,644)		FBG > 126mg/dL (n = 13,657)	
Age (years)	Female	4,619 (41.76%)	49 ± 14	4,208 (39.53%)	55 ± 12	5,670 (41.51%)	54 ± 12
	Male	6,438 (58.20%)	50 ± 14	6,434 (60.45%)	53 ± 13	7,986 (58.47%)	52 ± 13
FBG (mg/dL)		88.9 ± 8.4		111.9 ± 7.1		178.8 ± 57.6	
CRP (mg/dL)		0.94 ± 1.85		1.63 ± 4.34		1.7 ± 3.56	
WBC (× 10^9^/L)		8.12 ± 4.82		8.18 ± 3.24		8.59 ± 2.43	
Platelets (× 10^9^/L)		263 ± 75		263 ± 76		266 ± 78	
NLR		1.93 ± 1.04		1.96 ± 1.23		2.05 ± 1.59	
PLR		8.67 ± 4.23		8.7 ± 4.4		9.06 ± 5.4	
RBC (× 10^12^/L)		4.94 ± 0.67		4.97 ± 0.63		5.03 ± 0.665	
MCH (pg)		27.7 ± 2.9		27.6 ± 3.0		27.3 ± 3.0	
MCHC (g/dL)		32.6 ± 1.5		32.5 ± 1.5		32.5 ± 1.6	
MCV (fl)		84.6 ± 7.3		84.5 ± 7.5		83.7 ± 7.3	
HCT (%)		41.6 ± 5.4		42.0 ± 5.2		42.0 ± 5.3	
Hb (g/dL)		13.6 ± 1.9		13.6 ± 1.9		13.6 ± 1.9	

Mean values (± SD) for diagnostic biomarkers compared by glycemic levels under categorization by (A) HbA1c (n = 142,011) and (B) fasting blood glucose, FBG (n = 35,362).

*HbA1c* glycated hemoglobin type A1c, *FBG* fasting blood glucose, *WBC* white blood cells, *NLR* neutrophils to lymphocyte ratio, *PLR* platelets to Lymphocyte ratio.

Out of the 142,011 individuals who were tested for HbA1c, 60.94% had hyperglycemia (H), while 21.18% and 17.85% had borderline hyperglycemia (BH) and Normoglycemia(N), respectively, using American Diabetes Association (ADA) thresholds^14^. Higher rates of Hyperglycemia (53.45% vs 46.52%) and borderline hyperglycemia (54.81% vs 45.16%) were observed amongst males as compared to females (Table 1A), with no contrasting differences in the mean ages. Of the 35,362 individuals who got tested for FBG, 38.62% had hyperglycemia, 30.10% were borderline and 31.28% had normoglycemia. Males had higher rates of hyperglycemia (58.47%) as compared to females (41.51%). A similar trend was observed amongst males and females for borderline hyperglycemia (60.45% vs 39.53%), with similar mean ages, in the FBG classified sub-group (Table 1B). For both sub-groups (HbA1c and FBG), heightened mean expressions of CRP, WBC, Platelet, NLR, PLR, RBC, and HCT were observed in hyperglycemic state, whereas a negative trend was seen for RBC indices including MCV, MCH, and MCHC. Hb exhibited stable mean expression for all levels of glycemia (Table 1).

Together, our results show that with an increasing glycemia, CRP, WBC, Platelet, NLR, PLR, RBC, and HCT show an increasing trend whereas RBC indices tend to decrease.

### CRP, WBC, NLR, and PLR are significant discriminants for differentiating glycemic control

To evaluate the inflammatory response present in different glycemic conditions, we performed a multivariate analysis of variance, MANOVA, separately for two sub-groups, HbA1c (n = 2877) and FBG (n = 616). Our results using Pillai’s trace show that glycemic states (normal, borderline, and hyperglycemia) vary significantly with the five inflammatory markers (CRP, WBC, Platelet, NLR, PLR); group effect exhibiting F-ratio_HbA1c_ of 182.27 (p < 0.0001) and F-ratio _FBG_ of 37.249 (p < 0.0001) indicated by Pillai's trace.

To elucidate the effect of glycemia on inflammatory markers, a univariate one-way ANOVA test was employed. The results revealed that there was a statistically significant difference (p value < 0.0001) in the mean expression at least between 2 glycemic states for HbA1c (F = 1747.63), CRP (F = 19.226), WBC (F = 19.978), NLR (F = 20.952) and PLR (F = 13.93). Platelets were found to be statistically insignificant. The results are summarized in (Table 2A) for the HbA1c cohort. For the case of FBG cohort (F = 328.067), WBC (F = 13.359) and PLR (F = 8.607) were found to be significantly different (p-value < 0.001), along with CRP (F = 4.035, p = 0.018), platelets (F = 3.382, p = 0.035) and NLR (F = 8.607, p = 0.006) (Table 2B).

**Table 2:** Univariate tests of between-subject effects shown for the MANOVA models.

Dependent variable		Mean square	F-statistic	Sig
(A) ANOVA summary table of glycemic levels grouped by HbA1c				
Glycemic marker	**HbA1C (%)**	**2354.84**	**1747.639**	0.000**
Inflammatory markers	**CRP (mg/dL)**	**331.92**	**19.226**	0.000**
	**WBC (× 10**^**9**^**/L)**	**219.439**	**19.978**	0.000**
	Platelets (× 10^9^/L)	5336.485	0.669	0.512
	**NLR**	**134.942**	**20.952**	0.000**
	**PLR**	**963.174**	**13.937**	0.000**
(B) ANOVA summary table of glycemic levels grouped by fasting blood glucose (FBG)				
Glycemic marker	**FBG (mg/dL)**	**510,513.414**	**328.067**	0.000**
Inflammatory markers	**CRP (mg/dL)**	**44.862**	**4.035**	0.018*
	**WBC (× 10**^**9**^**/L)**	**80.035**	**13.359**	0.000**
	**Platelets (× 10**^**9**^**/L)**	**20,721.043**	**3.382**	0.035*
	**NLR**	**36.804**	**5.187**	0.006**
	**PLR**	**444.649**	**8.607**	0.000**
**(**C) ANOVA summary table of glycemic levels grouped by HbA1c, superimposed with RBC parameters				
Glycemic marker	**HbA1C (%)**	**25,066.905**	**16,440.736**	0.000**
Inflammatory markers	**WBC (× 10**^**9**^**/L)**	**1027.902**	**87.401**	0.000**
	**Platelets (× 10**^**9**^**/L)**	**23,483.169**	**3.069**	0.046*
	**NLR**	**22.106**	**7.164**	0.001**
	**PLR**	**240.999**	**4.575**	0.010*
RBC parameter	**RBC (× 10**^**12**^**/L)**	**37.418**	**81.71**	0.000**
	**MCH (pg)**	**1314.528**	**145.581**	0.000**
	**MCHC (g/dL)**	**121.352**	**61.533**	0.000**
	**MCV (fl)**	**8057.967**	**137.593**	0.000**
	HCT (%)	83.264	2.648	0.071
	Hb (g/dL)	3.353	0.844	0.43
**(**D) ANOVA summary table of glycemic levels grouped by fasting blood glucose (FBG) superimposed with RBC parameters				
Glycemic marker	**FBG (mg/dL)**	**6,296,398.395**	**4976.068**	0.000**
Inflammatory markers	**WBC (× 10**^**9**^**/L)**	**195.273**	**12.801**	0.000**
	Platelets (× 10^9^/L)	6086.779	1.001	0.368
	**NLR**	**31.022**	**15.65**	0.000**
	**PLR**	**285.861**	**11.844**	0.000**
RBC parameters	**RBC (× 10**^**12**^**/L)**	**5.481**	**13.102**	0.000**
	**MCH (pg)**	**40.867**	**4.785**	0.008**
	MCHC (g/dL)	0.74	0.384	0.681
	**MCV (fl)**	**356.418**	**6.463**	0.002**
	**HCT (%)**	**157.612**	**5.609**	0.004**
	**Hb (g/dL)**	**15.186**	**4.235**	0.015*

**Estimation of the individual ANO**VA results for Dependent variables (DVs) showing their dependency on independent variable (IV). (A) & (B) showing models that are fitted to contain data from five DVs (CRP, WBC, platelet, NLR and PLR) which are indicative of inflammation and one IV that is glycemic levels. (A) summarizes ANOVA results for HBA1c cohort and (B) for FBG cohort. (C) & (D) represents the model in which molecular markers (RBC, MCH, MCHC, MCV, HCT & Hb) are superimposed on inflammation (WBC, platelet, NLR and PLR) and model is fitted to have 10 DVs and 1 IV (HbA1c or FBG). (C) summarizes the ANOVA results for HbA1c cohort and (D) for FBG cohort.

The significant parameters and their respective p-values are highlighted in bold. *P < 0.05, **P < 0.01.

**Figure 1:**
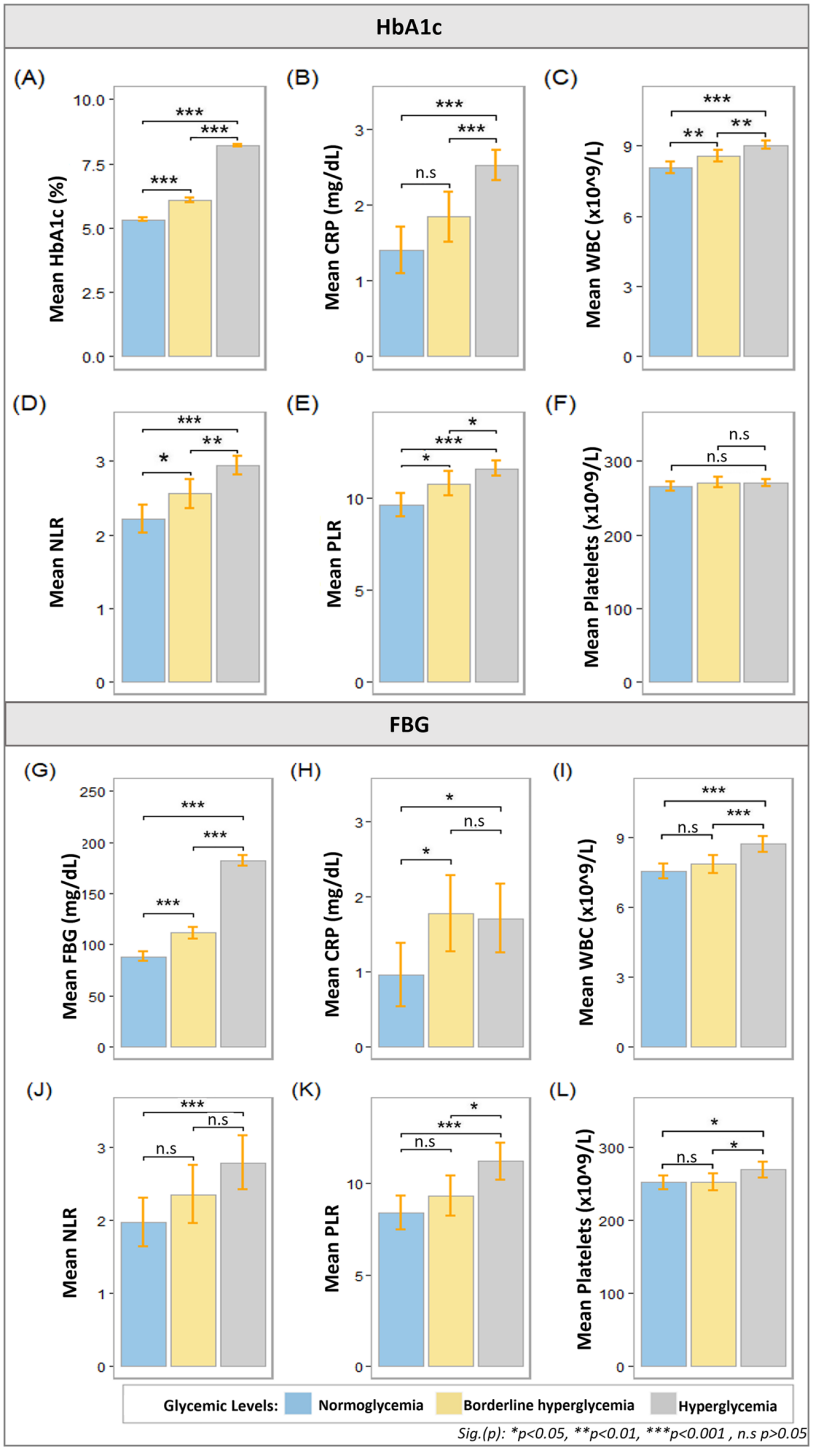
Graphical representation of multiple pairwise comparison between glycemic levels for HbA1c and FBG cohorts. HbA1c,FBG, CRP, WBC, NLR and PLR levels in the study sample are dependent on glycemic control. After a significant multivariate MANOVA model, we first assessed variations between groups of different levels of glycemia for HbA1c, FBG, CRP, WBC, NLR, PLR and Platelets (Table 2A, B). Least significant difference (LSD) method as post hoc test was employed to determine significant difference between groups and control error rate at α-level of 0.05. Graphs from (A-F) represents pairwise analysis for HbA1c cohorts and graphs from (G-L) represents pairwise results for FBG cohort. Length of Bar represents mean values while 95% CI are illustrated as error bars. Parameters (A-E) & (G-L) had significant Anova result at p-value < 0.001 and significant results for multiple comparisons are shown with: Sig.(p): *p < 0.05, **p < 0.01, ***p < 0.001 , ^#^p > 0.05.

Post-hoc LSD was employed for multiple pairwise comparisons while controlling the error rate at an alpha level of 0.05. LSD results for the HbA1c cohort revealed that HbA1c was significantly increased from N to BH (0.752 (95% CI 0.878 to 0.626) %, p < 0.0001), N to H (2.87 (95% CI, 2.97 to 2.77) %, p < 0.0001), and BH to H (2.116 (95% CI, 2.225 to 2.008) %, p < 0 0.0001). NLR was statistically significantly increased from N to BH (0.3443 (95% CI 0.6207 to 0.0678), p = 0.015), N to H (0.7294 (95% CI, 0.9556 to 0.5032), p < 0.0001), and BH to H (0.3852 (95% CI, 0.6233 to 0.1470), p = 0.002). PLR was also observed to be significantly increased from N to BH (1.1573 (95% CI 2.0628 to 0.2517), p = 0.012), N to H (1.9833 (95% CI, 2.7242 to 1.2425), p < 0.0001), and BH to H (0.8261 (95% CI, 1.6063 to 0.0459), p = 0.038). Notably, CRP was only significantly increased from N to H (1.1222 (95% CI, 1.4925 to 0.7519) mg/dl, p < 0.0001), and BH to H (0.6825 (95% CI, 1.0724 to 0.2925) mg/dl, p = 0.001). WBC, on the other hand, was statistically significantly increased from N to BH (0.4797 (95% CI, 0.8407 to 0.1187) × 109/L, p = 0.009), N to H (0.9372 (95% CI, 1.2326 to 0.6419), p < 0.0001) × 109/L, and BH to H (0.4575 (95% CI, 0.7686 to 0.1465) × 109/L, p = 0.004). Platelets were not found to significantly vary between any group. Results from the HbA1c cohort are provided in Fig. 1A–F and Supplementary Table S1.

The FBG cohort exhibited a significant increase in FBG from N to H (93.766 (95% CI, 101.126 to 86.407)mg/dl, p < 0.0001), and BH to H (70.713 (95% CI, 78.780 to 62.646) mg/dl, p < 0.0001), NLR was only statistically significantly increased from N to H 0.8150 (95% CI 1.3119 to 0.3181), PLR was only statistically significantly increased from N to H (2.8070 (95% CI, 4.1480 to 1.4661), p < 0.0001), and BH to H (1.8954 (95% CI, 3.3652 to 0.4255), p = 0.012). CRP was only statistically significantly increased from N to BH (0.8145 (95% CI 1.4699 to 0.1592) mg/dl, p = 0.015) mg/dl and N to H (0.7487 (95% CI, 1.3707 to 0.1266) mg/dl, p = 0.018). While, WBC was only statistically significantly increased from N to H (1.176 (95% CI, 1.6326 to 0.7193) × 10^9^/L, p < 0.0001), and BH to H (2.116 (95% CI, 1.3785 to -0.3774) × 10^9^/L, p = 0.001). Platelets were only significantly increased from N to H (17.50 (95% CI, 32.11 to 2.90) × 10^9^/L, p = 0.019), and BH to H (17.37 (95% CI, 33.38 to 1.37) × 10^9^/L, p = 0.033). Results are shown in Fig. 1G–L and (Supplementary Table S2).

### RBC superimposed on inflammation status as an augmented discriminator of glycemic control

To investigate the effect of glycemic status on RBCs status and inflammation, MANOVA was performed for two sub-cohorts i.e., HbA1c (n = 28,577) and FBG (n = 8376) for 11 dependent variables including inflammatory markers (WBCs, NLR, PLR, Platelets) as well as molecular markers (RBC count, Hb (hemoglobin), HCT, and RBC indices including MCV, MCH, and MCHC). Group effect estimated from multivariate test exhibits F-ratio_HbA1c_ of 960.097 (p < 0.0001) and F-ratio _FBG_ of 286.112 (p < 0.0001) as indicated by Pillai's trace. To further analyze the effect of glycemia on molecular and inflammatory markers, a univariate one-way ANOVA test was employed for the HbA1c cohort. ANOVA revealed that there was a statistically significant difference (p value < 0.0001) in mean expression between at least 2 states of glycemia for HbA1c (F = 16,440.736), WBC (F = 87.401), RBC (F = 81.710), MCH (F = 145.581), MCHC (F = 61.533), MCV (F = 137.593). NLR (F = 7.164), PLR (F = 4.575), and Platelets (F = 3.069) were also statistically significant but with a p-value < 0.05. HCT and Hb were found to be insignificant (Table 2C). For the FBG cohort, FBG (F = 4976.068), WBC (F = 12.801), NLR (F = 15.650), PLR (F = 11.844), RBC (F = 13.102) were significantly different (p-value < 0.0001), while MCH (F = 4.785), MCV (F = 6.463), HCT (F = 5.609) and Hb (F = 4.235) at p-value < 0.05, however, platelets and MCHC showed insignificant results (Table 2D).

**Figure 2:**
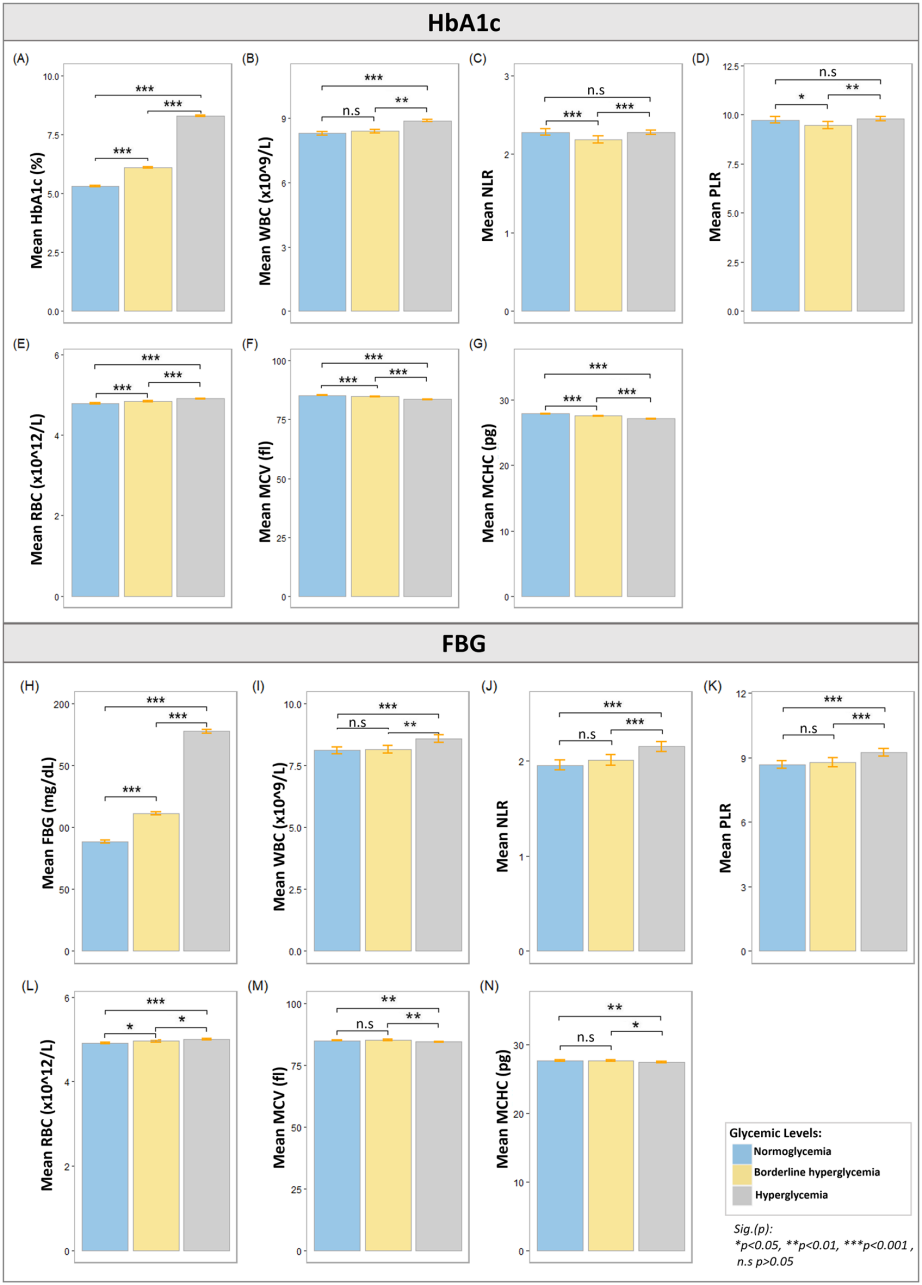
Graphical representation of multiple pairwise comparison between glycemic levels for HbA1c and FBG cohorts for joint model. HbA1c, FBG, WBC, NLR and PLR, RBC, MCH, MCHC and MCV levels in the study sample are dependent on glycemic status. After a significant multivariate MANOVA model, we first assessed variations between groups of different levels of glycemia for HbA1c, FBG, CRP, WBC, NLR, PLR, platelets, RBC, MCH, MCHC and MCV (Table 2C, D). Least significant difference (LSD) method as post hoc test was employed to determine significant difference between groups and control error rate at α-level of 0.05. Graphs from (**A–G**) represent pairwise analysis for HbA1c cohorts and graphs from (**H–N**) represent pairwise results for FBG cohort. Length of bar represents mean values while 95% CI are illustrated as error bars. Parameters (**A–N**) had significant ANOVA result at p-value < 0.001 and significant results for multiple comparisons are shown with: Sig.(p): *p < 0.05, **p < 0.01, ***p < 0.001, ^#^p > 0.05.

Post-hoc LSD for significant ANOVAs for multiple pairwise comparisons for the HbA1c cohort revealed that HbA1c was significantly increased from N to BH (0.776 (95% CI, 0.818 to 0.733) %, p < 0.0001), N to H (2.978 (95% CI, 3.013 to 2.943) %, p < 0.0001), and BH to H (2.203 (95% CI, 2.239 to 2.166) %, p < 0.0001). NLR statistically significantly decreased from N to BH (−0.0971 (95% CI, −0.0362 to −0.1579), p = 0.002), and statistically significantly increased from BH to H (0.0957 (95% CI, 0.1477 to 0.0437), p < 0.0001). PLR only statistically significantly decreased from N to BH (−0.2769 (95% CI, −0.0254 to −0.5284), p = 0.031), and significantly increased from BH to H (0.3288 (95% CI, 0.5437 to 0.1139) %, p = 0.003). WBC was only statistically significantly increased from N to H (0.5840 (95% CI, 0.6818 to 0.4863) × 10^9^/L, p < 0.0001), and BH to H (0.4796 (95% CI, 0.5811 to 0.3780) × 10^9^/L, p < 0.0001). RBC was statistically significantly increased from N to BH (0.0466 (95% CI, 0.0700 to 0.0231) × 10^12^/L, p < 0.0001), N to H (0.120 (95% CI, 0.1393 to 0.1007) × 10^12^/L, p < 0.0001), and BH to H (0.0735 (95% CI, 0.0935 to 0.0534) × 10^12^/L, p < 0.0001). Platelets were only significantly increased from BH to H (2.83 (95% CI, 5.42 to 0.24) × 10^9^/L, p = 0.032). MCH was statistically significantly decreased from N to BH (−0.329 (95% CI, −5.42 to −0.24) pg, p < 0.0001), N to H (−0.724 (95% CI, −0.618 to −0.829) pg, p < 0.0001), and BH to H (−0.395 (95% CI, −0.286 to −0.504) pg, p < 0.0001). MCHC was statistically significantly increased from N to BH (0.202 (95% CI, 0.153 to 0.250) g/dl, p < 0.0001), N to H (0.222 (95% CI, 0.182 to 0.262) g/dl, p < 0.0001). MCV was only statistically significantly decreased from N to BH (−0.202 (95% CI, −0.153 to −0.250) fl, p < 0.0001) and N to H (−0.222 (95% CI, −0.182 to −0.262) fl, p < 0.0001). Results for the HbA1c cohort are given in Fig. 2A–F and Supplementary Table S3. Multiple pairwise test results for the FBG cohort showed FBG to be increased significantly from N to BH (23.046 (95% CI, 24.944 to 21.147) mg/dl p < 0.0001), N to H (89.322 (95% CI, 91.131 to 87.512) mg/dl, p < 0.0001), and BH to H (66.276 (95% CI, 68.191 to 64.362) mg/dl, p < 0.0001). NLR was only statistically significantly increased from N to H (0.1981 (95% CI, 0.2697 to 0.1265), p < 0.0001), and BH to H (0.1476 (95% CI, 0.2233 to 0.0718), p < 0.0001). PLR was also only statistically significantly increased from N to H (0.5924 (95% CI, 0.8423 to 0.3425), p < 0.0001), and BH to H (0.4729 (95% CI, 0.7373 to 0.2085), p < 0.0001). WBC was also only statistically significantly increased from N to H (0.4693 (95% CI, 0.6679 to 0.2706) × 10^9^/L, p < 0.00001), and BH to H (0.4309 (95% CI, 0.6411 to 0.2207 ) × 10^9^/L, p < 0.0001). RBC was statistically significantly increased from N to BH (0.0435 (95% CI, 0.0781 to 0.0090) × 10^12^/L, p = 0.013), N to H (0.0859 (95% CI, 0.1188 to 0.0530) × 10^12^/L, p < 0.0001), and BH to H ( 0.0424 (95% CI, 0.0772 to 0.0076) × 10^12^/L, p = 0.017). MCH was significantly decreased only from N to H (-0.211 (95% CI, -0.063 to -0.360) pg, p = 0.005), and BH to H (-0.202 (95% CI, -0.045 to -0.359) pg, p < 0.012). MCV was also only statistically significantly decreased from N to H (-0.568 (95% CI,- 0.190 to -0.945) fl, p < 0.0001), and BH to H (-0.658 (95% CI, -0.258 to -1.057) fl, p < 0.0001). HCT was only statistically significantly increased from N to BH (0.378 (95% CI, 0.661 to 0.095) %, p = 0.009) and N to H (0.422 (95% CI, 0.692 to 0.153) %, p = 0.002). Hb was also statistically significantly increased from N to BH (0.115 (95% CI, 0.216 to 0.014) g/dl, p = 0.026), N to H (0.133 (95% CI, 0.229 to 0.036) g/dl, p = 0.007). Results are summarized graphically for the FBG cohort in Fig. 2G–L and Supplementary Table S4.

### A predictive model of non-specific inflammatory markers for estimation of dysglycemia

**Figure 3:**
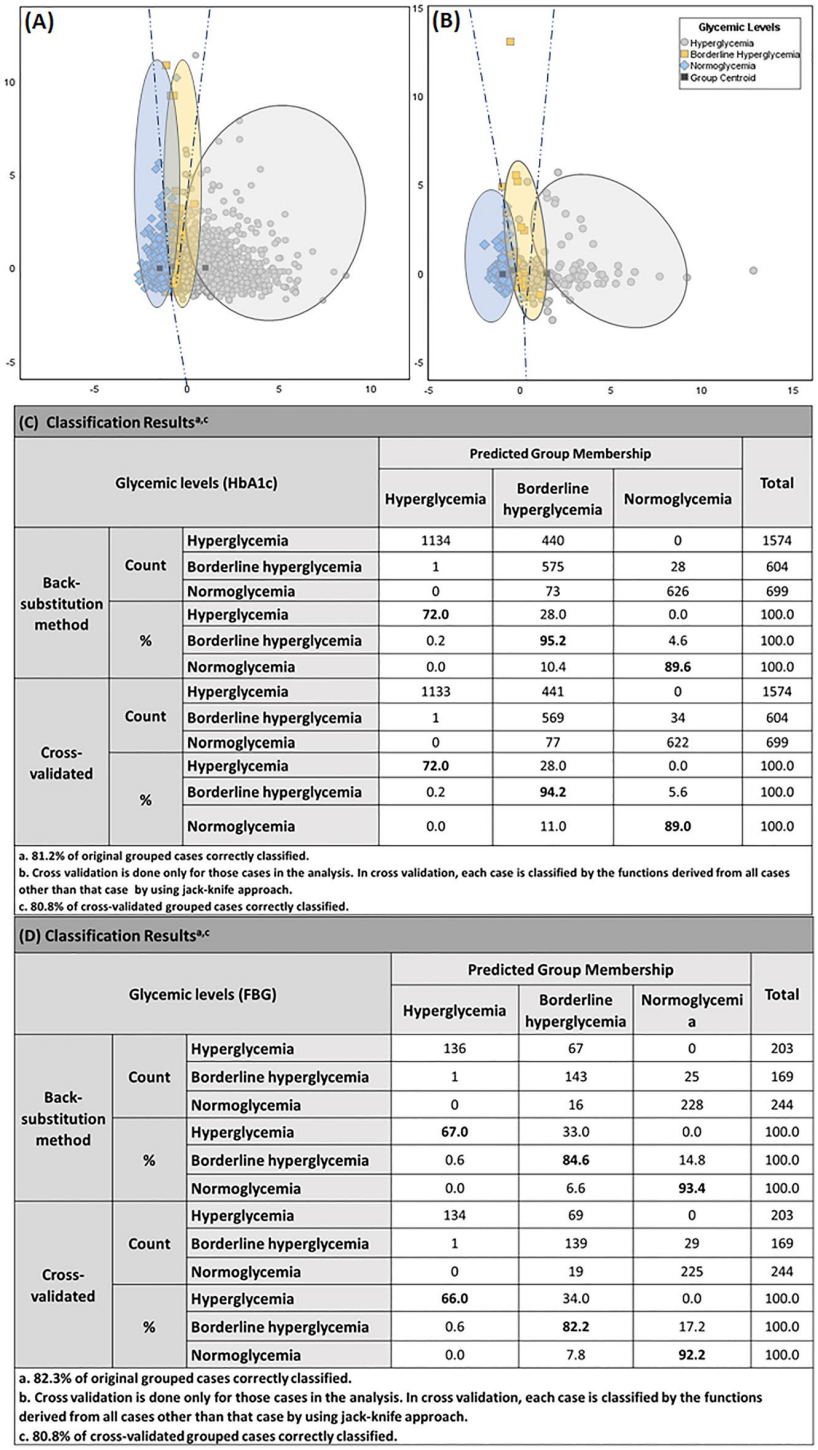
Linear discriminant analysis (LDA) results for predictive model consisting of inflammatory markers for estimation of dysglycemia. (**A**) The HbA1c cohort Combined plot of the discriminant functions generated from 5 inflammatory parameters. Each data point represents a single reading in the study sample. The plot illustrates close but distinctive clustering and separation of hyperglycemia (grey circles), borderline hyperglycemia (yellow squares) and normoglycemia (blue diamonds). Dark grey square represents group centroid. Dashed dark blue line depicts linear decision boundary. (**C**) Classification results for back substitution method for HbA1c with an accuracy of 81.2% original grouped cases correctly classified and after jackknife cross validation shows 80.8% accurate results. Linear discriminant analysis (LDA) results for FBG cohort (**B**) Combined plot of the discriminant functions generated from 5 inflammatory predictors. Dark grey square represents group centroid. Dashed dark blue line depicts linear decision boundary. (**D**) Classification results for back substitution method for FBG cohort with an accuracy of 82.3% original grouped cases correctly classified and after jackknife cross validation shows 80.8% accurate results. Individual group classification is highlighted in bold, model for HbA1c is showing highest accuracy in predicting borderline hyperglycemia whereas in the case of FBG showing normoglycemia to be grouped with maximum accuracy of 93.4 of the original grouped cases followed by borderline hyperglycemia which is showing 84.6% accuracy.

Linear discriminant analysis (LDA) was used to develop a glycemic prediction model comprising six clinical parameters out of which five were inflammatory markers (CRP, WBC, Platelet, NLR, PLR), where CRP is an indicator of chronic inflammation and one was glycemic indicator (HbA1c or FBG) to differentiate between different states of glycemia (hyperglycemia, borderline hyperglycemia, and normoglycemia). Results are displayed in Fig. 3A, B for HbA1c and FBG cohorts, respectively. Using the variances from all the values, two discriminant functions were derived, which accounted for 100% of the variance. For both the HbA1c and FBG cohort, the first canonical discriminant function contributed substantially towards the total variance in the dataset with more than 99% variance with a canonical correlation of 0.7, at a significance value p < 0.001 in both cases.

The classification discriminant functions (DF0, DF1, and DF2) were therefore generated based on the estimation of corresponding β values (Table 3A) for the HbA1c cohort and (Table 3B) for the FBG cohort.

1 $$\mathbf{P}\left(\mathbf{y}=0|\mathbf{x}\right)\mathbf{H}\mathbf{b}\mathbf{A}1\mathbf{c}=-36.39+\left(5.996*HbA1c\right)+\left(3.305*NLR\right)-\left(1.159*PLR\right)-(0.153*CRP)+(0.519*WBC)+(0.077*Platelets)$$

2 $$\mathbf{P}\left(\mathbf{y}=1|\mathbf{x}\right)\mathbf{H}\mathbf{b}\mathbf{A}1\mathbf{c}= -24.944+\left(4.428*HbA1c\right)+\left(3.198*NLR\right)-\left(1.128*PLR\right)-(0.157*CRP)+(0.504*WBC)+(0.076*Platelets)$$

3 $$\mathbf{P}(\mathbf{y}=2|\mathbf{x})\mathbf{H}\mathbf{b}\mathbf{A}1\mathbf{c}=- 21.507+\left(3.877*HbA1c\right)+\left(3.173*NLR\right)-\left(1.13*PLR\right)-\left(0.161*CRP\right)+\left(0.481*WBC\right)+\left(0.076*Platelets\right)$$

4 $$\mathbf{P}\left(\mathbf{y}=0|\mathbf{x}\right)\mathbf{F}\mathbf{B}\mathbf{G}=-26.006+\left(0.118*FBG\right)+\left(3.624*NLR \right)-\left(1.545*PLR \right)-(0.149*CRP )+(1.081*WBC )+(0.098*Platelets )$$

5 $$\mathbf{P}\left(\mathbf{y}=1|\mathbf{x}\right)\mathbf{F}\mathbf{B}\mathbf{G}= -18.083+\left(0.072*FBG\right)+\left(3.769*NLR\right)-\left(1.627*PLR\right)-(0.06*CRP )+(0.97*WBC)+(0.098*Platelets)$$

6 $$\mathbf{P}\left(\mathbf{y}=2|\mathbf{x}\right)\mathbf{F}\mathbf{B}\mathbf{G}= -16.423+\left(0.057*FBG\right)+\left(3.744*NLR\right)-\left(1.625*PLR\right)-\left(0.116*CRP\right)+\left(0.966*WBC\right)+\left(0.098*Platelets\right)$$

where, y = 0 means belongingness of the hyperglycemia subset, y = 1 belongingness to borderline hyperglycemia, and y = 2, belongingness to normoglycemia.

**Table 3:** Linear discriminant functions for the predictive modelling.

Predictors		Wilks' Lambda	p-value	Hyperglycemia	Borderline hyperglycemia	Normoglycemia
(A) Classification function coefficients for HbA1c cohort^a,b^						
Glycemic marker	HbA1C	0.451	< 0.001	5.996	4.428	3.877
Inflammatory markers	CRP	0.987	< 0.001	−0.153	−0.157	−0.161
	WBC	0.986	< 0.001	0.519	0.504	0.481
	Platelet	1	< 0.001	0.077	0.076	0.076
	NLR	0.986	< 0.001	3.305	3.198	3.173
	PLR	0.99	< 0.001	−1.159	−1.128	−1.13
(B) Classification function coefficients for FBG cohort^a,b^						
Glycemic marker	FBG	0.483	< 0.001	0.118	0.072	0.057
Inflammatory markers	CRP	0.987	0.018	−0.149	−0.06	−0.116
	WBC	0.958	< 0.001	1.081	0.97	0.966
	Platelet	0.989	0.035	0.098	0.098	0.098
	NLR	0.983	0.006	3.624	3.769	3.744
	PLR	0.973	< 0.001	−1.545	−1.627	−1.625
(C) Classification function coefficients for HbA1c cohort^a,c^						
Glycemic marker	HbA1C	0.471	< 0.001	5.089	3.703	3.228
Inflammatory markers	WBC	0.994	< 0.001	0.479	0.443	0.437
	NLR	1	< 0.001	−0.115	−0.166	−0.142
	PLR	1	< 0.001	0.61	0.624	0.627
RBC indices	RBC	0.996	< 0.001	20.083	20.257	20.24
	MCH	0.99	< 0.001	−3.485	−3.44	−3.316
	MCV	0.99	< 0.001	3.566	3.561	3.518
(D) Classification function coefficients for FBG cohort^a,c^						
Glycemic marker	FBG	0.455	< 0.001	0.135	0.084	0.067
Inflammatory markers	WBC	0.997	< 0.001	0.393	0.364	0.36
	NLR	0.998	< 0.001	−2.144	−2.174	−2.207
	PLR	0.998	< 0.001	1.596	1.601	1.609
RBC indices	RBC	0.996	< 0.001	23.788	23.851	23.817
	MCH	0.998	< 0.001	−3.172	−3.152	−3.104
	MCV	0.997	< 0.001	3.746	3.741	3.72

Predictive model for chronic dysglycemia using inflammatory predictors. (A) Classification function coefficients for the HbA1c cohort. (B) Classification function coefficients for FBG cohort. Joint predictive model for chronic dysglycemia using inflammatory markers in combination with molecular markers. (C) Classification function coefficients of joint model for HbA1c cohort. (D) Classification function coefficients of joint model for FBG cohort.

Fisher's linear discriminant functions.

^a^Model significant at p < 0.001.

^b^Predictive model of non-specific inflammatory markers for estimation of chronic Dysglycemia.

^c^Joint predictive model of inflammatory markers in combination with erythrocytes status for estimation of chronic dysglycemia.

### A joint predictive model of inflammatory markers in combination with erythrocytes status for estimation of dysglycemia

**Figure 4:**
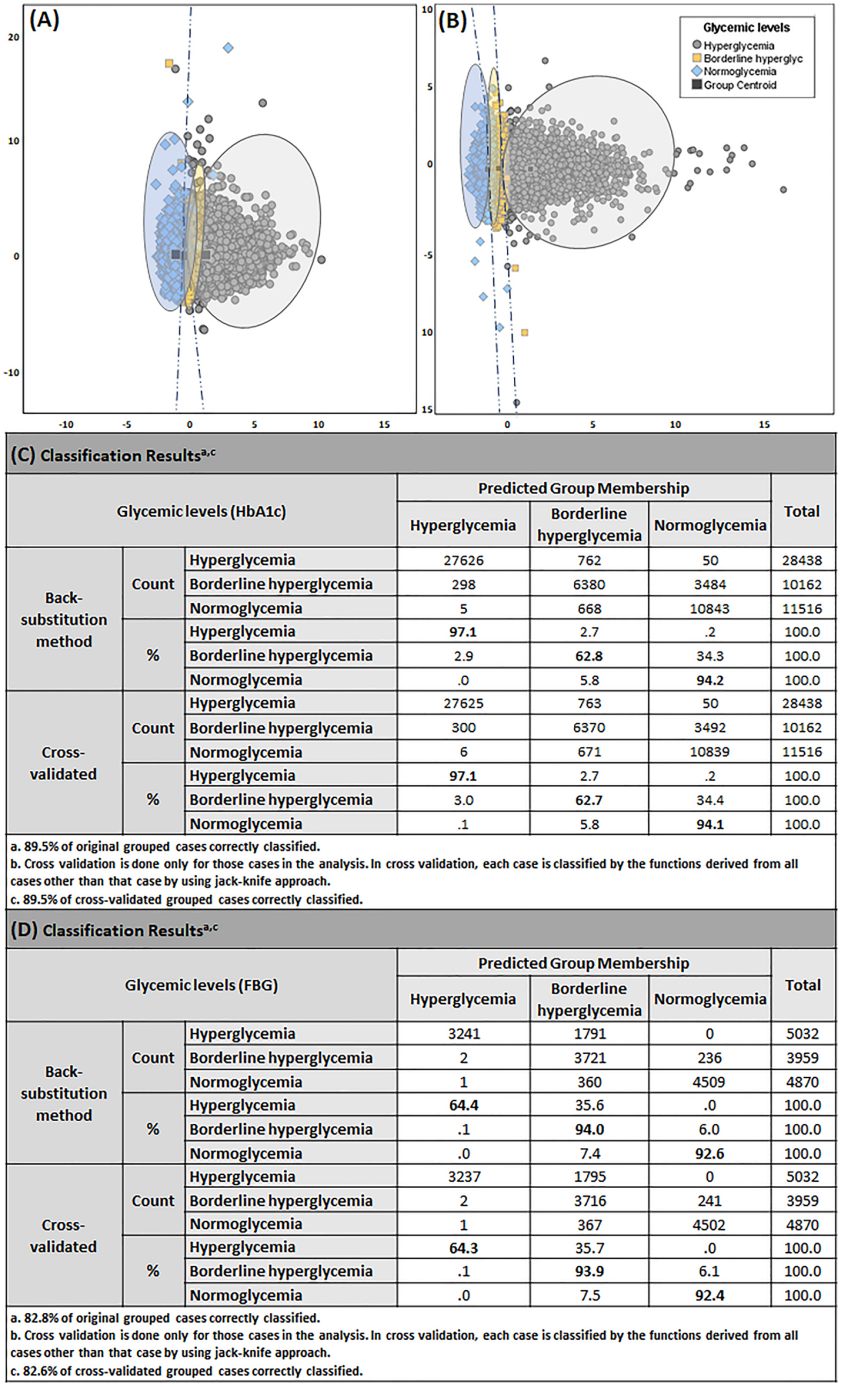
Linear discriminant analysis (LDA) results for joint predictive model of inflammatory markers in combination with erthrocytes status for estimation of dysglycemia (**A**) shows the HbA1c cohort Combined plot of the discriminant functions generated from 3 inflammatory parameters (NLR, PLR and WBC count) predictors of erythrocytes status (RBC count, MCV and MCH) and glycemic status indicator (HbA1c or FBG). Each data point represents a single reading in the study sample. The plot illustrates close but distinctive clustering and separation of hyperglycemia (grey circles), borderline hyperglycemia (yellow squares) and normoglycemia (blue diamonds). The dark grey square represents the group centroid. Dashed dark blue line depicts linear decision boundary. (**C**) Classification results for back substitution method for HbA1c cohort with an accuracy of 89.5% original grouped cases correctly classified and after jackknife cross validation shows 89.5% accurate results. Linear discriminant analysis (LDA) results for FBG cohort. (**B**) Combined plot of the discriminant functions generated from inflammatory and RBC predictors. Each data point represents a single reading in the study sample. The plot illustrates close but distinctive clustering and separation of hyperglycemia (grey circles), borderline hyperglycemia (yellow squares) and normoglycemia (blue diamonds). The dark grey square represents group centroid. Dashed dark blue line depicts linear decision boundary. (**D**) Classification results for back substitution method for FBG cohort with an accuracy of 82.8% original grouped cases correctly classified and after jackknife cross validation shows 82.6% accurate results. Individual group classification is highlighted in bold, model for HbA1c is showing highest accuracy in predicting hyperglycemia (97.1%) whereas in the case of FBG showing borderline hyperglycemia to be grouped with maximum accuracy of 94% of the original grouped cases followed by normoglycemia which is showing 92.6% accuracy.

We applied LDA to develop a glycemic predictive model with enhanced accuracy by integration of the significant DVs, concluded from the outcomes of individual ANOVAs (Table 2C, D). Predictors of inflammation (NLR, PLR, and WBC count), predictors of erythrocytes status (RBC count, MCV, and MCH), and glycemic status indicator (HbA1c or FBG) were used to differentiate between different states of glycemia. Results are displayed in Fig. 4A, B for HbA1c and FBG cohorts, respectively. Two discriminant functions were derived, which accounted for 100% of the variance. For the HbA1c cohort (n = 50,116) first canonical discriminant function majorly contributed towards the total variance in the dataset with 99.9% variance and canonical correlation of 0.729, however for the FBG cohort (n = 13,861) first canonical discriminant accounted for 100% variance with the canonical correlation of 0.739, at a significance p-value < 0.001 in both cases. The classification discriminant functions (DF0, DF1, and DF2) were therefore generated based on the estimation of corresponding β values (Table 3C) for the HbA1c cohort and (Table 3D) for the FBG cohort.

7 $$\mathbf{P}\left(\mathbf{y}=0|\mathbf{x}\right)\mathbf{H}\mathbf{b}\mathbf{A}1\mathbf{c}=-177.133+\left(5.089*HbA1c\right)+\left(0.479*WBC\right)-\left(0.115*NLR\right)+\left(0.610*PLR\right)+\left(20.083*RBC\right)- \left(3.485*MCH\right)+ \left(3.566*MCV\right)$$

8 $$\mathbf{P}\left(\mathbf{y}=1|\mathbf{x}\right)\mathbf{H}\mathbf{b}\mathbf{A}1\mathbf{c}= -169.510+\left(3.703*HbA1c\right)+\left(0.443*WBC\right)-\left(0.166*NLR\right)+\left(0.624*PLR\right)+\left(20.257*RBC\right)- \left(3.44*MCH\right)+ \left(3.561*MCV\right)$$

9 $$\mathbf{P}\left(\mathbf{y}=2|\mathbf{x}\right)\mathbf{H}\mathbf{b}\mathbf{A}1\mathbf{c}=-166.396+\left(3.228*HbA1c\right)+\left(0.437*WBC\right)-\left(0.142*NLR\right)+\left(0.627*PLR\right)+\left(20.240*RBC\right)- \left(3.316*MCH\right)+ \left(3.518*MCV\right)$$

10 $$\mathbf{P}\left(\mathbf{y}=0|\mathbf{x}\right)\mathbf{F}\mathbf{B}\mathbf{G}=-193.222+\left(0.135*FBG\right)+\left(0.393*WBC\right)-\left(2.144*NLR\right)+\left(1.596*PLR\right)+\left(23.788*RBC\right)- \left(3.172*MCH\right)+ \left(3.746*MCV\right)$$

11 $$\mathbf{P}\left(\mathbf{y}=1|\mathbf{x}\right)\mathbf{F}\mathbf{B}\mathbf{G} =-186.011+\left(0.084*FBG\right)+\left(0.364*WBC\right)-\left(2.174*NLR\right)+\left(1.601*PLR\right)+\left(23.851*RBC\right)- \left(3.152*MCH\right)+ \left(3.741*MCV\right)$$

12 $$\mathbf{P}\left(\mathbf{y}=2|\mathbf{x}\right)\mathbf{F}\mathbf{B}\mathbf{G} =-183.696+\left(0.067*FBG\right)+\left(0.360*WBC\right)-\left(2.207*NLR\right)+\left(1.609*PLR\right)+\left(23.817*RBC\right)- \left(3.104*MCH\right)+ \left(3.720*MCV\right)$$

where, y = 0 means belongingness of the hyperglycemia subset, y = 1 belongingness to borderline hyperglycemia, and y = 2, belongingness to normoglycemia.

### An evaluation of model accuracy to predict dysglycemia from inflammatory markers

**Figure 5:**
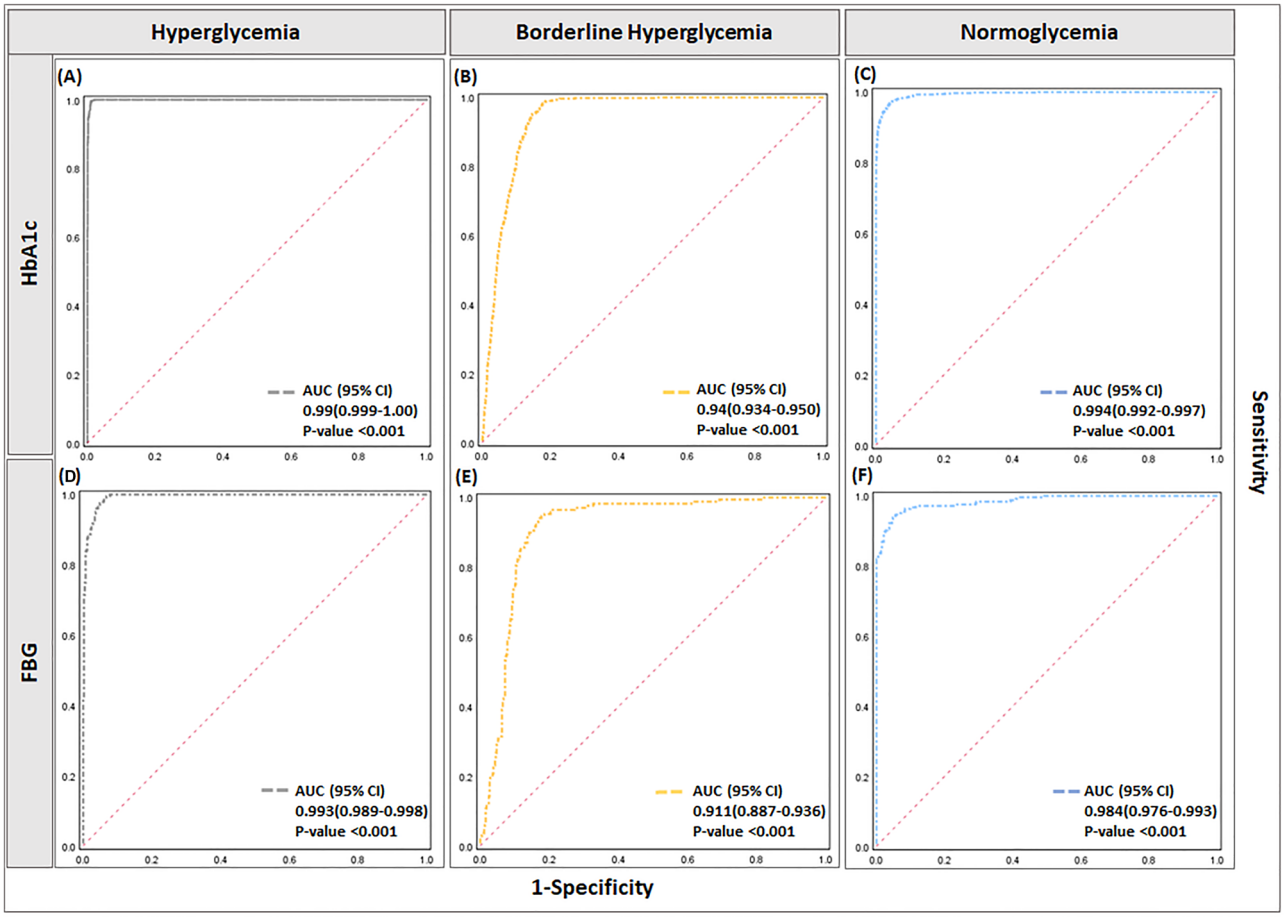
ROC (*ROC curve receiver-operating-characteristics curve*) analysis for inflammatory predictive model for estimation of dysglycemia in HbA1c and FBG cohort. (**A,B**) Illustrates ROC curves for the HbA1c cohort, (**A**) AUC for hyperglycemia is 0.99(95% CI: 0.999–1.00), (**B**) AUC for borderline hyperglycemia is 0.94(95% CI: 0.934–0.950) and (**C**) AUC for normoglycemia is 0.994 (95% CI: 0.992–0.997). (**D–F**) Illustrates ROC curves for the FBG cohort, (**A**) AUC for hyperglycemia is 0.993(95% CI: 0.989–0.998), (**B**) AUC for borderline hyperglycemia is 0.911 (95% CI: 0.887–0.936) and (**C**) AUC for normoglycemia is 0.984(95% CI: 0.976–0.993). All results significant at p-value < 0.001.

Discriminant classification results showed good separations of the three glycemic states for both cohorts with an accuracy of greater than 80% (Fig. 3C, D). Classification results for the HbA1c cohort (Fig. 3C) showed that the back substitution method can classify hyperglycemia with a correct discrimination proportion of 72%; for the borderline hyperglycemia subset, 95.2% and for normoglycemia cases with a correct discrimination proportion of 89.6%. Moreover, classification results for the FBG cohort (Fig. 3D) showed correct group membership of about 67% for hyperglycemia, 84.6% for borderline hyperglycemia, and for normoglycemia cases, it was 93.4%. For both cohorts, borderline hyperglycemia had the highest correct discrimination proportion results. To further evaluate the stability of the model discriminant functions, Jackknife cross-validation was employed, which showed almost similar classification accuracy for both the HbA1c (80.8%) and FBG (80.8%) LDA models. ROC (receiver-operating-characteristics curve) analysis of the model computed AUC and 95% CI values for each glycemic type for both cohorts. The model exhibited a strong diagnostic value for glycemic state with all ROCs showing AUC above 0.9 (p < 0.0001, Fig. 5).

### An evaluation of joint model accuracy in the prediction of dysglycemia

**Figure 6:**
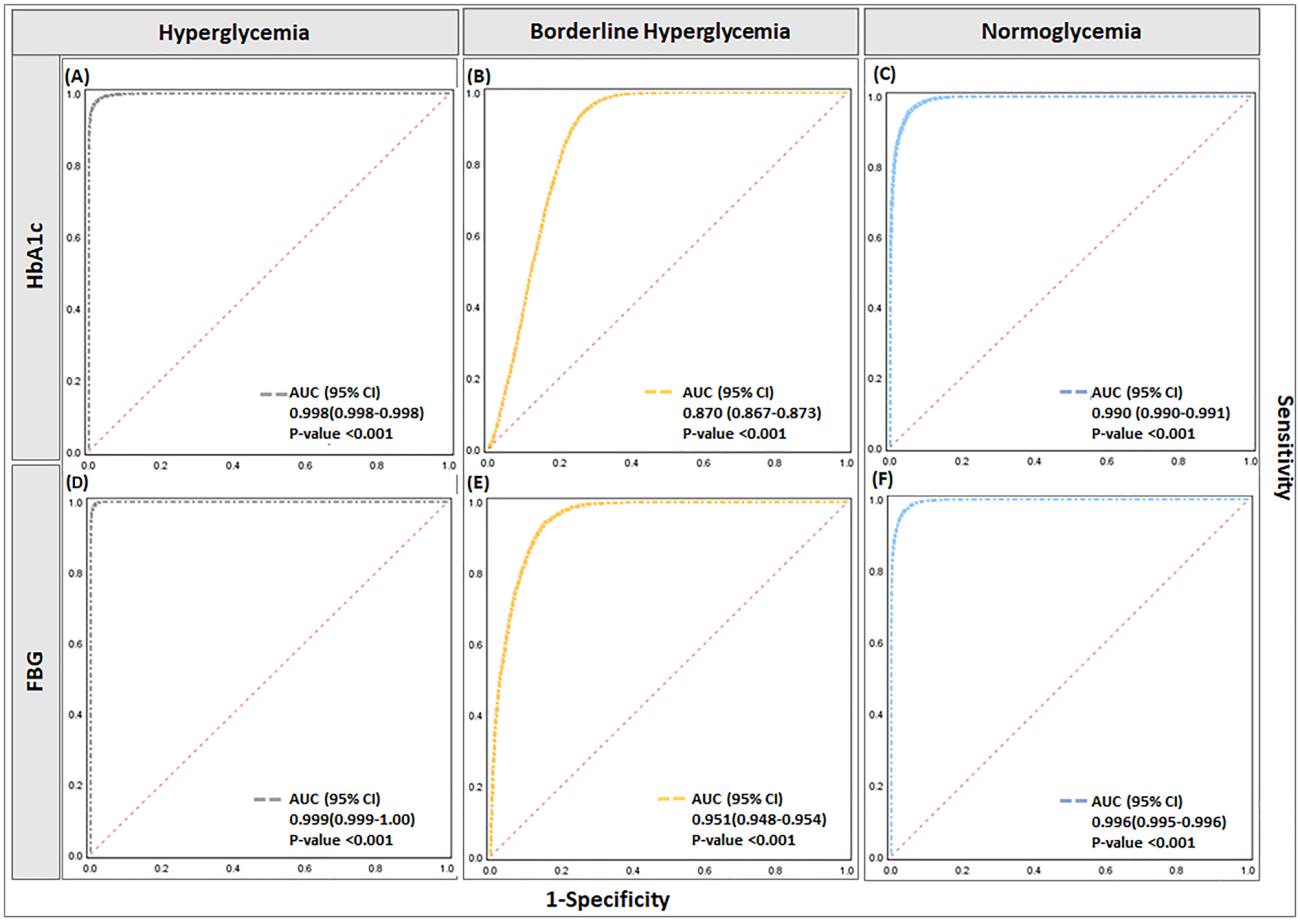
ROC (*receiver-operating-characteristics curve*) analysis joint predictive model of inflammatory markers in combination with erthrocytes status for estimation of dysglycemia in HbA1c and FBG cohort. (**A,B**) Illustrates ROC curves for the HbA1c cohort, (**A**) AUC for hyperglycemia is 0.998(95% CI: 0.998–0.998), (**B**) AUC for borderline hyperglycemia is 0.870 (95% CI: 0.867–0.873) and (**C**) AUC for normoglycemia is 0.990 (95% CI: 0.990–0.991). (**D–F**) Illustrates ROC curves for the FBG cohort, (**A**) AUC for hyperglycemia is 0.999 (95% CI: 0.999–1.00), (**B**) AUC for borderline hyperglycemia is 0.951 (95% CI: 0.948–0.954) and (**C**) AUC for normoglycemia is 0.996(95% CI: 0.995–0.996). All results significant at p-value < 0.001.

Upon integration of significant candidates from RBC parameters with the inflammatory markers in the HbA1c model, the overall accuracy increased from 81.2 to 89.5% (Fig. 4C), thus providing evidence of a strong discriminatory value of parameter superimposition. Notably, no difference was observed for the FBG cohort (82.3% vs 82.8%) (Fig. 4D). Interestingly, the joint HbA1c cohort model showed 25% improvement in accuracy for hyperglycemia (72% vs 97.1%). The FBG cohort showed an increment of 10% in the predictive ability for borderline hyperglycemia (84% vs 94%). Jackknife cross-validation results were comparable with the classification accuracy for both HbA1c (89.5%) and FBG (82.6%) LDA models. Furthermore, ROC assessment established the diagnostic specificity and sensitivity of the joint model (p < 0.0001) with all ROC AUCs above 0.9 except for the HbA1c cohort in predicting borderline hyperglycemia (AUC = 0.87). Results are illustrated in Fig. 6.

### Low cost and high accuracy risk fingerprinting of dysglycemia

The prediction accuracy for the inflammatory (“M1”: Eqs. 1–3, “M2”: Eqs. 4–6) and joint (“M3”: Eqs. 7–9, “M4”: Eqs. 10–12) model for both HbA1c and FBG are provided in Table 4. M1 provided the highest discrimination proportion of 95.2% for predicting borderline hyperglycemia but had the highest cost (~ $20). M4 had 94% prediction accuracy for borderline hyperglycemia with the lowest price at just ~ $5. Importantly, M3 had the highest discriminatory power for the correct classification of hyperglycemia (97.1%) and normoglycemia (94.2%) cases. In the case of the FBG cohort, the overall predictive capacity for both models M2 vs M4 (82.3% vs 82.8%) was comparable but at 2.5 times the price difference ($12 vs. $5). Overall, amongst all the reported models, M3 provided the highest cumulative predictive accuracy of 89.5% for hyperglycemia, borderline hyperglycemia, and normal cases. In conclusion, M3 and M4 models could be utilized for population-level screening programs and by clinicians for predicting hyperglycemia and borderline hyperglycemia at a lower cost.

**Table 4:** Cumulative results for overall model accuracy and their individual group (glycemic outcomes) classification results with respective cost estimates.

Diagnostics markers	Diagnostic tests		% Predictive accuracy				Average cost^a^
Molecular biomarker			Hyperglycemia (%)	Borderline hyperglycemia (%)	Normoglycemia (%)	Overall model (%)	
Inflammation	M1	HbA1c + CBC + CRP	72	95.20	89.60	81.20	PKR4900 ($20)
	M2	FBG + CBC + CRP	67	84.60	93.40	82.30	PKR3000 ($12)
Inflammation + RBC status	M3	HbA1c + CBC	97.10	62.80	94.20	89.50	PKR3000 ($12)
	M4	FBG + CBC	64.40	94.00	92.60	82.80	PKR1200 ($5)

M1: Model 1, M2: Model 2, M3: Model 3, M4: Model 4.

^a^Is an average of test prices from three largest labs in Pakistan for 2023.

## Discussion

In this study, we have investigated the interplay between inflammation, RBC parameters, and hyperglycemia by employing clinical diagnostics of CBC, CRP, HbA1c, and FBG (fasting blood glucose) towards developing a predictive model of glycemic outcomes. Our results show that variations in inflammatory profile in addition to derangements in RBC indices can be formulated into a powerful predictive tool for measuring dysglycemia with considerable precision. This proposed approach can be particularly useful in population-level risk fingerprinting of DM and evaluation of patient’s health outcomes concerning hyperglycemia.

Aberrations in the immune system are central to the incidence and progression of DM^17,18^. Modern research has also furnished evidence on the role of inflammation in the onset of pro-inflammatory pathways in insulin production which then lead to the initiation of metabolic disorders including DM^38^. CRP (c-reactive protein) is one such chronic inflammatory marker that has a direct association with the risk of type 2 diabetes^21,38^. The current study is consistent with literature reports that an increasing trend in CRP was observed in hyperglycemia, in comparison to normal or borderline state (Table 1A, B). We, therefore, propose a glycemic status (normoglycemia, borderline-hyperglycmeia, hyperglycemia) prediction model by using glycemic indicators in concert with other routine clinical diagnostics (CBC and CRP). For that, we started off by developing a MANOVA model (categorical glycemic status as independent, diagnostic variables as dependents) to evaluate the significance of multivariate association between different parameters. Follow-up univariate individual ANOVAs and multiple pairwise comparisons (Fig. 1) also indicate CRP to be significantly different amongst three levels, for both HbA1c and FBG cohorts. WBC—a component of CBC, is also a nonspecific indicator of inflammation and is reported to be a predictor in the pathogenesis of diabetes^20,25^. Furthermore, NLR and PLR were estimated from diagnostic data and used as inflammatory indices to associate hyperglycemia with inflammation. NLR and PLR are well reported to have predictive power for DM ^27–29^ with studies reporting NLR and PLR values to be higher in DM^39,40^. The results of the current study are in agreement (Table 2) showing the levels of WBCs, NLR, and PLR to be significantly different among the three glycemic groups and significantly raised from normoglycemia to hyperglycemia (Figs. 1 and 2).

Moreover, other hematological counters in CBC including RBCs can provide insight into the state of glycation. Since RBCs are sensitive to changes in plasma composition, therefore, long-term hyperglycemia alters RBC physiology and associated indices^32,35,41^. In the current study, a significant difference was observed in RBC count, MCV, and MCH (p-value < 0.01) between different glycemic states (Supplementary Table S3 and S4). A negative trend was seen for MCV and MCH with the increase in HbA1c and FBG for both cohorts (Fig. 2E–G, L–N) which are in line with studies that report a negative correlation between HbA1c and MCV and MCH^42,43^. Therefore, the employment of RBC-related indicators can also provide a useful reference for the diagnosis and prognosis of diabetes. Furthermore, several studies report that Hb variants in hemoglobinopathies and anemias interfere with the accurate measurement of HbA1c^44–46^. In the current study, the superimposition of RBC indices on inflammation improved the discriminatory power of the model for three states of glycemia. Hence, proving to be a versatile tool in parallel with HbA1c and FBG, for effective assessment of hyperglycemia.

According to the molecular and inflammatory descriptors in the proposed models, each biomarker contributed in predicting different glycemic outcomes. In addition, we attempted to establish reliable LDA models which could reveal the underlying dimensionality of the data while specifying the contribution of each parameter to the glycemic status group classification. Taken together, the combined results from both the post-hoc univariate and multivariate analyses exhibited significant group separation in the multivariate space. This provided evidence that the selected parameters manifested multivariate characteristics, which made it imperative to employ a multivariate follow-up technique to decipher the latent dependence within the dataset towards formulating a glycemic status prediction model. This approach is also in light of prior literature ^47–50^ which highligts follow-up employment of LDA—a post-hoc method of choice after MANOVA for predictive modelling. Note that LDA uniquely served both as an interpretive technique along with graphical representation of the classified data.

These models inculcated the influence of inflammation and derangement in various hematological parameters in the prediction of three states of glycemia, as independent variables. Moreover, it could be projected that the establishment of the discriminant functions that are based on inflammatory descriptors provided significant predictive power but the integration of RBC indices enhanced the prediction accuracy by 8.3% (from 81.2 to 89.5%). The improved model shows the full valuation of HbA1c (%) being explained by six types of biomarkers which consequently enhanced the discriminatory power of the model. The presented discriminant equations (Eqs. 7–9) for the models could be used as an in silico screening tool for the prediction of glycemic outcomes. Different models could be opted for according to the type and budget of the screening program (Table 4).

To compare the performance and utility of supervised learning methods, we also evaluated other ML techniques including Multinomial Logistic Regression (MLR), Multilayer Perceptron (MLP), and K-Nearest Neighbors. For the case of MLR, the model demonstrated that one predictor (HbA1c or FBG) significantly outweighs others in terms of coefficient magnitude. MLP, on the other hand, though learned the complex nonlinear relationship between predictors and outcomes, was adversely affected by one input neuron (HbA1c or FBG) as revealed by feature importance analysis. KNN model also exhibited limited efficacy in overcoming this issue. This underperformance of models can be attributed to several factors which makes it essential to acknowledge that the choice between classification techniques is contingent upon not just on the parametric and non-parametric assumptions but also on the characteristics of the dataset, and the presence of multicollinearity ^51^ among variables. In line with this, existing literature suggests that LDA, under certain conditions and in comparison to other techniques like logistic regression, multinomial logistic regression, random forests, support-vector machines, and the K-nearest neighbor algorithm, performs better in group membership prediction^52,53^. Tharwat et al^54^ conducted experiments with different datasets to investigate the effect of eigenvectors in LDA space on the robustness of the extracted features for classification accuracy. Likewise, Tao Li^55^ suggested in their experimental investigation that LDA proves to be a fast and accurate option for the multi-class classification problems.

This study demonstrates that LDA modeling can aid in the cost-effective screening of hyperglycemia and borderline hyperglycemia using data from simple routine lab diagnostic tests. Further, in conjunction with normal examination procedures, this tool could assist in better diagnosis and management of diabetes.

In terms of limitations, this study suffers from the unavailability of the DM status amongst hyperglycemic sub-groups. Also, the current study utilized clinical data of walk-in visitors in clinical diagnostic laboratories, which includes both patients and healthy individuals. Therefore, as a future extension, we propose to deploy the reported models in clinical settings where diagnosis information could be utilized for fine-tuning the models towards the development of higher-accuracy screening models.

## Conclusion

The increasing burden of diabetes makes it imperative to investigate prophylactic interventions in comparison to treatments, through early detection of diabetes. Reliance on FBG alone might result in under-reporting as patients may be asymptomatic or adhering to strict dietary regimens besides the usage of medications before testing. For that, clinical diagnostics data can be used to screen patients for detecting the early onset of diabetes, onwards investigations, as well as disease management. The proposed model provides a low-cost platform with considerable accuracy for detection of hyperglycemia which in succession would have the capacity to improve the quality of life by checking the treatment cost and comorbidities. Together, the smart screening tool could assist in informing DM investigations, and its management along with the prevention of clinical complications related to chronic hyperglycemia.

### Supplementary Information

Supplementary Tables.

## Data Availability

The dataset generated and analysed during the current study is not publicly available due ongoing data analysis on the same dataset as an extension of another research project but is available from the corresponding author on reasonable request.
