# Supplementary material for: Fingerprinting hyperglycemia using predictive modelling approach based on low-cost routine CBC and CRP diagnostics
Source: Sci Rep. 2024 Jan 11;14:1090. doi: 10.1038/s41598-023-44623-4 (PMC10784542; doi:10.1038/s41598-023-44623-4)
Supplement: Supplementary file 1 — Supplementary Tables. [file 41598_2023_44623_MOESM1_ESM.docx]

**Fingerprinting Hyperglycemia using Predictive Modelling Approach based on Low-Cost Routine CBC and CRP Diagnostics**

Amna Tahir^1^, Kashif Asghar^2^, Waqas Shafiq^3^, Hijab Batool^4^, Dilawar Khan^4^, Omar Chughtai^4^, Safee Ullah Chaudhary^1, *^

^1^ Biomedical Informatics & Engineering Research Laboratory, Department of Life Sciences, Syed Babar Ali School of Science and Engineering, Lahore University of Management Sciences, Lahore, Pakistan,

^2^ Basic Science Department, Shaukat Khanum Memorial Cancer Hospital and Research Center, Lahore, Pakistan,

^3^ Department of Internal Medicine, Shaukat Khanum Memorial Cancer Hospital and Research Center, Lahore, Pakistan,

^4^ Chughtai Institute of Pathology, Lahore, Pakistan.

* Corresponding Author

**Keywords**: Hyperglycemia, Diabetes Mellitus (DM), Complete Blood Count (CBC), HbA1c, Fasting Blood Glucose (FBG), Red Blood Cells (RBC), Inflammation

**Supplementary Table S1: LSD Multiple Comparisons for HbA1c cohort including inflammatory parameters.**

| **LSD Multiple Comparisons for HbA1c cohort** | | | | | | | |
| --- | --- | --- | --- | --- | --- | --- | --- |
| **Dependent Variable** | **(I) Glycemic levels** | **(J) Glycemic levels** | **Mean Difference (I-J)** | **Std. Error** | **Sig.** | **95% Confidence Interval** | |
|  |  |  |  |  |  | **Lower Bound** | **Upper Bound** |
| **HbA1C** | **Hyperglycemia** | **Borderline hyperglycemia** | 2.116* | .0556 | .000 | 2.008 | 2.225 |
|  |  | **Normoglycemia** | 2.868* | .0528 | .000 | 2.765 | 2.972 |
|  | **Borderline hyperglycemia** | **Hyperglycemia** | -2.116* | .0556 | .000 | -2.225 | -2.008 |
|  |  | **Normoglycemia** | .752* | .0645 | .000 | .626 | .878 |
|  | **Normoglycemia** | **Hyperglycemia** | -2.868* | .0528 | .000 | -2.972 | -2.765 |
|  |  | **Borderline hyperglycemia** | -.752* | .0645 | .000 | -.878 | -.626 |
| **NLR** | **Hyperglycemia** | **Borderline hyperglycemia** | .3852* | .12147 | .002 | .1470 | .6233 |
|  |  | **Normoglycemia** | .7294* | .11535 | .000 | .5032 | .9556 |
|  | **Borderline hyperglycemia** | **Hyperglycemia** | -.3852* | .12147 | .002 | -.6233 | -.1470 |
|  |  | **Normoglycemia** | .3443* | .14099 | .015 | .0678 | .6207 |
|  | **Normoglycemia** | **Hyperglycemia** | -.7294* | .11535 | .000 | -.9556 | -.5032 |
|  |  | **Borderline hyperglycemia** | -.3443* | .14099 | .015 | -.6207 | -.0678 |
| **PLR** | **Hyperglycemia** | **Borderline hyperglycemia** | .8261* | .39790 | .038 | .0459 | 1.6063 |
|  |  | **Normoglycemia** | 1.9833* | .37785 | .000 | 1.2425 | 2.7242 |
|  | **Borderline hyperglycemia** | **Hyperglycemia** | -.8261* | .39790 | .038 | -1.6063 | -.0459 |
|  |  | **Normoglycemia** | 1.1573* | .46183 | .012 | .2517 | 2.0628 |
|  | **Normoglycemia** | **Hyperglycemia** | -1.9833* | .37785 | .000 | -2.7242 | -1.2425 |
|  |  | **Borderline hyperglycemia** | -1.1573* | .46183 | .012 | -2.0628 | -.2517 |
| **CRP** | **Hyperglycemia** | **Borderline hyperglycemia** | .6825* | .19887 | .001 | .2925 | 1.0724 |
|  |  | **Normoglycemia** | 1.1222* | .18885 | .000 | .7519 | 1.4925 |
|  | **Borderline hyperglycemia** | **Hyperglycemia** | -.6825* | .19887 | .001 | -1.0724 | -.2925 |
|  |  | **Normoglycemia** | .4397 | .23083 | .057 | -.0129 | .8923 |
|  | **Normoglycemia** | **Hyperglycemia** | -1.1222* | .18885 | .000 | -1.4925 | -.7519 |
|  |  | **Borderline hyperglycemia** | -.4397 | .23083 | .057 | -.8923 | .0129 |
| **WBC** | **Hyperglycemia** | **Borderline hyperglycemia** | .4575* | .15863 | .004 | .1465 | .7686 |
|  |  | **Normoglycemia** | .9372* | .15064 | .000 | .6419 | 1.2326 |
|  | **Borderline hyperglycemia** | **Hyperglycemia** | -.4575* | .15863 | .004 | -.7686 | -.1465 |
|  |  | **Normoglycemia** | .4797* | .18412 | .009 | .1187 | .8407 |
|  | **Normoglycemia** | **Hyperglycemia** | -.9372* | .15064 | .000 | -1.2326 | -.6419 |
|  |  | **Borderline hyperglycemia** | -.4797* | .18412 | .009 | -.8407 | -.1187 |
| **Platelet** | **Hyperglycemia** | **Borderline hyperglycemia** | -.27 | 4.275 | .950 | -8.65 | 8.12 |
|  |  | **Normoglycemia** | 4.41 | 4.060 | .277 | -3.55 | 12.37 |
|  | **Borderline hyperglycemia** | **Hyperglycemia** | .27 | 4.275 | .950 | -8.12 | 8.65 |
|  |  | **Normoglycemia** | 4.68 | 4.962 | .346 | -5.05 | 14.41 |
|  | **Normoglycemia** | **Hyperglycemia** | -4.41 | 4.060 | .277 | -12.37 | 3.55 |
|  |  | **Borderline hyperglycemia** | -4.68 | 4.962 | .346 | -14.41 | 5.05 |
| Based on observed means.  The error term is Mean Square(Error) = 7978.101.  *The mean difference is significant at the .05 level. | | | | | | | |

**Supplementary Table S2: LSD Multiple Comparisons for FBG cohort including inflammatory parameters.**

| **LSD Multiple Comparisons for FBG cohort** | | | | | | | |
| --- | --- | --- | --- | --- | --- | --- | --- |
| **Dependent Variable** | **(I) Glycemic levels** | **(J) Glycemic levels** | **Mean Difference (I-J)** | **Std. Error** | **Sig.** | **95% Confidence Interval** | |
|  |  |  |  |  |  | **Lower Bound** | **Upper Bound** |
| **NLR** | **Hyperglycemia** | **Borderline hyperglycemia** | .4358 | .27736 | .117 | -.1089 | .9805 |
|  |  | **Normoglycemia** | .8150^*^ | .25304 | .001 | .3181 | 1.3119 |
|  | **Borderline hyperglycemia** | **Hyperglycemia** | -.4358 | .27736 | .117 | -.9805 | .1089 |
|  |  | **Normoglycemia** | .3791 | .26657 | .155 | -.1444 | .9026 |
|  | **Normoglycemia** | **Hyperglycemia** | -.8150^*^ | .25304 | .001 | -1.3119 | -.3181 |
|  |  | **Borderline hyperglycemia** | -.3791 | .26657 | .155 | -.9026 | .1444 |
| **PLR** | **Hyperglycemia** | **Borderline hyperglycemia** | 1.8954^*^ | .74845 | .012 | .4255 | 3.3652 |
|  |  | **Normoglycemia** | 2.8070^*^ | .68280 | .000 | 1.4661 | 4.1480 |
|  | **Borderline hyperglycemia** | **Hyperglycemia** | -1.8954^*^ | .74845 | .012 | -3.3652 | -.4255 |
|  |  | **Normoglycemia** | .9117 | .71932 | .205 | -.5010 | 2.3243 |
|  | **Normoglycemia** | **Hyperglycemia** | -2.8070^*^ | .68280 | .000 | -4.1480 | -1.4661 |
|  |  | **Borderline hyperglycemia** | -.9117 | .71932 | .205 | -2.3243 | .5010 |
| **CRP** | **Hyperglycemia** | **Borderline hyperglycemia** | -.0659 | .34722 | .850 | -.7478 | .6160 |
|  |  | **Normoglycemia** | .7487^*^ | .31677 | .018 | .1266 | 1.3707 |
|  | **Borderline hyperglycemia** | **Hyperglycemia** | .0659 | .34722 | .850 | -.6160 | .7478 |
|  |  | **Normoglycemia** | .8145^*^ | .33371 | .015 | .1592 | 1.4699 |
|  | **Normoglycemia** | **Hyperglycemia** | -.7487^*^ | .31677 | .018 | -1.3707 | -.1266 |
|  |  | **Borderline hyperglycemia** | -.8145^*^ | .33371 | .015 | -1.4699 | -.1592 |
| **WBC** | **Hyperglycemia** | **Borderline hyperglycemia** | .8780^*^ | .25488 | .001 | .3774 | 1.3785 |
|  |  | **Normoglycemia** | 1.1760^*^ | .23252 | .000 | .7193 | 1.6326 |
|  | **Borderline hyperglycemia** | **Hyperglycemia** | -.8780^*^ | .25488 | .001 | -1.3785 | -.3774 |
|  |  | **Normoglycemia** | .2980 | .24496 | .224 | -.1831 | .7790 |
|  | **Normoglycemia** | **Hyperglycemia** | -1.1760^*^ | .23252 | .000 | -1.6326 | -.7193 |
|  |  | **Borderline hyperglycemia** | -.2980 | .24496 | .224 | -.7790 | .1831 |
| **Platelet** | **Hyperglycemia** | **Borderline hyperglycemia** | 17.37^*^ | 8.151 | .033 | 1.37 | 33.38 |
|  |  | **Normoglycemia** | 17.50^*^ | 7.436 | .019 | 2.90 | 32.11 |
|  | **Borderline hyperglycemia** | **Hyperglycemia** | -17.37^*^ | 8.151 | .033 | -33.38 | -1.37 |
|  |  | **Normoglycemia** | .13 | 7.834 | .987 | -15.26 | 15.51 |
|  | **Normoglycemia** | **Hyperglycemia** | -17.50^*^ | 7.436 | .019 | -32.11 | -2.90 |
|  |  | **Borderline hyperglycemia** | -.13 | 7.834 | .987 | -15.51 | 15.26 |
| **FBG** | **Hyperglycemia** | **Borderline hyperglycemia** | 70.713^*^ | 4.1077 | .000 | 62.646 | 78.780 |
|  |  | **Normoglycemia** | 93.766^*^ | 3.7474 | .000 | 86.407 | 101.126 |
|  | **Borderline hyperglycemia** | **Hyperglycemia** | -70.713^*^ | 4.1077 | .000 | -78.780 | -62.646 |
|  |  | **Normoglycemia** | 23.053^*^ | 3.9478 | .000 | 15.300 | 30.806 |
|  | **Normoglycemia** | **Hyperglycemia** | -93.766^*^ | 3.7474 | .000 | -101.126 | -86.407 |
|  |  | **Borderline hyperglycemia** | -23.053^*^ | 3.9478 | .000 | -30.806 | -15.300 |
| Based on observed means.  The error term is Mean Square(Error) = 1556.126.  *The mean difference is significant at the .05 level. | | | | | | | |

**Supplementary Table S3: LSD Multiple Comparisons for HbA1c cohort including inflammatory and RBC parameters.**

| **LSD Multiple Comparisons for HbA1c cohort** | | | | | | | | | | | |
| --- | --- | --- | --- | --- | --- | --- | --- | --- | --- | --- | --- |
| **Dependent Variable** | **(I) Glycemic levels** | **(J) Glycemic levels** | **Mean Difference (I-J)** | | **Std. Error** | | **Sig.** | | **95% Confidence Interval** | | |
|  |  |  |  |  |  |  |  |  | **Lower Bound** | | **Upper Bound** |
| **HbA1C** | **Hyperglycemia** | **Borderline hyperglycemia** | | 2.203^*^ | | .0187 | | .000 | | 2.166 | 2.239 |
|  |  | **Normoglycemia** | | 2.978^*^ | | .0180 | | .000 | | 2.943 | 3.013 |
|  | **Borderline hyperglycemia** | **Hyperglycemia** | | -2.203^*^ | | .0187 | | .000 | | -2.239 | -2.166 |
|  |  | **Normoglycemia** | | .776^*^ | | .0218 | | .000 | | .733 | .818 |
|  | **Normoglycemia** | **Hyperglycemia** | | -2.978^*^ | | .0180 | | .000 | | -3.013 | -2.943 |
|  |  | **Borderline hyperglycemia** | | -.776^*^ | | .0218 | | .000 | | -.818 | -.733 |
| **NLR** | **Hyperglycemia** | **Borderline hyperglycemia** | | .0957^*^ | | .02654 | | .000 | | .0437 | .1477 |
|  |  | **Normoglycemia** | | -.0014 | | .02554 | | .958 | | -.0514 | .0487 |
|  | **Borderline hyperglycemia** | **Hyperglycemia** | | -.0957^*^ | | .02654 | | .000 | | -.1477 | -.0437 |
|  |  | **Normoglycemia** | | -.0971^*^ | | .03105 | | .002 | | -.1579 | -.0362 |
|  | **Normoglycemia** | **Hyperglycemia** | | .0014 | | .02554 | | .958 | | -.0487 | .0514 |
|  |  | **Borderline hyperglycemia** | | .0971^*^ | | .03105 | | .002 | | .0362 | .1579 |
| **PLR** | **Hyperglycemia** | **Borderline hyperglycemia** | | .3288^*^ | | .10964 | | .003 | | .1139 | .5437 |
|  |  | **Normoglycemia** | | .0519 | | .10553 | | .623 | | -.1549 | .2587 |
|  | **Borderline hyperglycemia** | **Hyperglycemia** | | -.3288^*^ | | .10964 | | .003 | | -.5437 | -.1139 |
|  |  | **Normoglycemia** | | -.2769^*^ | | .12831 | | .031 | | -.5284 | -.0254 |
|  | **Normoglycemia**  **Hyperglycemia** | **Hyperglycemia** | | -.0519 | | .10553 | | .623 | | -.2587 | .1549 |
|  |  | **Borderline hyperglycemia** | | .2769^*^ | | .12831 | | .031 | | .0254 | .5284 |
|  |  | **Borderline hyperglycemia** | | .2769 | | .13048 | | .096 | | -.0327 | .5866 |
| **WBC** | **Hyperglycemia** | **Borderline hyperglycemia** | | .4796^*^ | | .05180 | | .000 | | .3780 | .5811 |
|  |  | **Normoglycemia** | | .5840^*^ | | .04986 | | .000 | | .4863 | .6818 |
|  | **Borderline hyperglycemia** | **Hyperglycemia** | | -.4796^*^ | | .05180 | | .000 | | -.5811 | -.3780 |
|  |  | **Normoglycemia** | | .1045 | | .06062 | | .085 | | -.0144 | .2233 |
|  | **Normoglycemia**  **Hyperglycemia** | **Hyperglycemia** | | -.5840^*^ | | .04986 | | .000 | | -.6818 | -.4863 |
|  |  | **Borderline hyperglycemia** | | -.1045 | | .06062 | | .085 | | -.2233 | .0144 |
| **RBC** | **Hyperglycemia** | **Borderline hyperglycemia** | | .0735^*^ | | .01022 | | .000 | | .0534 | .0935 |
|  |  | **Normoglycemia** | | .1200^*^ | | .00984 | | .000 | | .1007 | .1393 |
|  | **Borderline hyperglycemia** | **Hyperglycemia** | | -.0735^*^ | | .01022 | | .000 | | -.0935 | -.0534 |
|  |  | **Normoglycemia** | | .0466^*^ | | .01196 | | .000 | | .0231 | .0700 |
|  | **Normoglycemia**  **Hyperglycemia** | **Hyperglycemia** | | -.1200^*^ | | .00984 | | .000 | | -.1393 | -.1007 |
|  |  | **Borderline hyperglycemia** | | -.0466^*^ | | .01196 | | .000 | | -.0700 | -.0231 |
|  |  | **Borderline hyperglycemia** | | -.0466^*^ | | .01201 | | .000 | | -.0751 | -.0180 |
| **Platelet** | **Borderline hyperglycemia** | **Hyperglycemia** | | 2.83^*^ | | 1.321 | | .032 | | .24 | 5.42 |
|  |  | **Normoglycemia** | | 2.31 | | 1.272 | | .070 | | -.18 | 4.80 |
|  | **Normoglycemia** | **Hyperglycemia** | | -2.83^*^ | | 1.321 | | .032 | | -5.42 | -.24 |
|  |  | **Borderline hyperglycemia** | | -.52 | | 1.546 | | .737 | | -3.55 | 2.51 |
|  | **Hyperglycemia** | **Borderline hyperglycemia** | | -2.31 | | 1.272 | | .070 | | -4.80 | .18 |
|  |  | **Normoglycemia** | | .52 | | 1.546 | | .737 | | -2.51 | 3.55 |
| **MCH** | **Borderline hyperglycemia** | **Hyperglycemia** | | -.395^*^ | | .0454 | | .000 | | -.484 | -.306 |
|  |  | **Normoglycemia** | | -.724^*^ | | .0437 | | .000 | | -.809 | -.638 |
|  | **Normoglycemia** | **Hyperglycemia** | | .395^*^ | | .0454 | | .000 | | .306 | .484 |
|  |  | **Borderline hyperglycemia** | | -.329^*^ | | .0531 | | .000 | | -.433 | -.225 |
|  | **Hyperglycemia** | **Borderline hyperglycemia** | | .724^*^ | | .0437 | | .000 | | .638 | .809 |
|  |  | **Normoglycemia** | | .329^*^ | | .0531 | | .000 | | .225 | .433 |
| **MCHC** | **Borderline hyperglycemia** | **Hyperglycemia** | | -.020 | | .0212 | | .351 | | -.061 | .022 |
|  |  | **Normoglycemia** | | -.222^*^ | | .0204 | | .000 | | -.262 | -.182 |
|  | **Normoglycemia** | **Hyperglycemia** | | .020 | | .0212 | | .351 | | -.022 | .061 |
|  |  | **Borderline hyperglycemia** | | -.202^*^ | | .0248 | | .000 | | -.250 | -.153 |
|  | **Hyperglycemia** | **Borderline hyperglycemia** | | .222^*^ | | .0204 | | .000 | | .182 | .262 |
|  |  | **Normoglycemia** | | .202^*^ | | .0248 | | .000 | | .153 | .250 |
| **MCV** | **Borderline hyperglycemia** | **Hyperglycemia** | | -1.165^*^ | | .1156 | | .000 | | -1.391 | -.938 |
|  |  | **Normoglycemia** | | -1.728^*^ | | .1113 | | .000 | | -1.946 | -1.510 |
|  | **Normoglycemia** | **Hyperglycemia** | | 1.165^*^ | | .1156 | | .000 | | .938 | 1.391 |
|  |  | **Borderline hyperglycemia** | | -.563^*^ | | .1353 | | .000 | | -.828 | -.298 |
|  | **Hyperglycemia** | **Borderline hyperglycemia** | | 1.728^*^ | | .1113 | | .000 | | 1.510 | 1.946 |
|  |  | **Normoglycemia** | | .563^*^ | | .1353 | | .000 | | .298 | .828 |
| **HCT** | **Borderline hyperglycemia** | **Hyperglycemia** | | .070 | | .0847 | | .407 | | -.096 | .236 |
|  |  | **Normoglycemia** | | .187^*^ | | .0815 | | .022 | | .027 | .347 |
|  | **Normoglycemia** | **Hyperglycemia** | | -.070 | | .0847 | | .407 | | -.236 | .096 |
|  |  | **Borderline hyperglycemia** | | .117 | | .0991 | | .238 | | -.077 | .311 |
|  | **Hyperglycemia** | **Borderline hyperglycemia** | | -.187^*^ | | .0815 | | .022 | | -.347 | -.027 |
|  |  | **Normoglycemia** | | -.117 | | .0991 | | .238 | | -.311 | .077 |
| **Hb** | **Borderline hyperglycemia** | **Hyperglycemia** | | .011 | | .0301 | | .720 | | -.048 | .070 |
|  |  | **Normoglycemia** | | -.032 | | .0290 | | .275 | | -.088 | .025 |
|  | **Normoglycemia** | **Hyperglycemia** | | -.011 | | .0301 | | .720 | | -.070 | .048 |
|  |  | **Borderline hyperglycemia** | | -.042 | | .0352 | | .229 | | -.112 | .027 |
|  | **Hyperglycemia** | **Borderline hyperglycemia** | | .032 | | .0290 | | .275 | | -.025 | .088 |
|  |  | **Normoglycemia** | | .042 | | .0352 | | .229 | | -.027 | .112 |
| Based on observed means.  The error term is Mean Square(Error) = 3.975.  * The mean difference is significant at the .05 level. | | | | | | | | | | | |

**Supplementary Table S4: LSD Multiple Comparisons for FBG cohort including inflammatory and RBC parameters.**

| **LSD Multiple Comparisons for FBG cohort** | | | | | | | |
| --- | --- | --- | --- | --- | --- | --- | --- |
| **Dependent Variable** | **(I) Glycemic levels** | **(J) Glycemic levels** | **Mean Difference (I-J)** | **Std. Error** | **Sig.** | **95% Confidence Interval** | |
|  |  |  |  |  |  | **Lower Bound** | **Upper Bound** |
| **NLR** | **Hyperglycemia** | **Borderline hyperglycemia** | .1476^*^ | .03866 | .000 | .0718 | .2233 |
|  |  | **Normoglycemia** | .1981^*^ | .03654 | .000 | .1265 | .2697 |
|  | **Borderline hyperglycemia** | **Hyperglycemia** | -.1476^*^ | .03866 | .000 | -.2233 | -.0718 |
|  |  | **Normoglycemia** | .0506 | .03833 | .187 | -.0246 | .1257 |
|  | **Normoglycemia** | **Hyperglycemia** | -.1981^*^ | .03654 | .000 | -.2697 | -.1265 |
|  |  | **Borderline hyperglycemia** | -.0506 | .03833 | .187 | -.1257 | .0246 |
| **PLR** | **Hyperglycemia** | **Borderline hyperglycemia** | .4729^*^ | .13489 | .000 | .2085 | .7373 |
|  |  | **Normoglycemia** | .5924^*^ | .12749 | .000 | .3425 | .8423 |
|  | **Borderline hyperglycemia** | **Hyperglycemia** | -.4729^*^ | .13489 | .000 | -.7373 | -.2085 |
|  |  | **Normoglycemia** | .1195 | .13375 | .372 | -.1427 | .3817 |
|  | **Normoglycemia** | **Hyperglycemia** | -.5924^*^ | .12749 | .000 | -.8423 | -.3425 |
|  |  | **Borderline hyperglycemia** | -.1195 | .13375 | .372 | -.3817 | .1427 |
| **WBC** | **Hyperglycemia** | **Borderline hyperglycemia** | .4309^*^ | .10724 | .000 | .2207 | .6411 |
|  |  | **Normoglycemia** | .4693^*^ | .10135 | .000 | .2706 | .6679 |
|  | **Borderline hyperglycemia** | **Hyperglycemia** | -.4309^*^ | .10724 | .000 | -.6411 | -.2207 |
|  |  | **Normoglycemia** | .0383 | .10633 | .718 | -.1701 | .2468 |
|  | **Normoglycemia**  **Hyperglycemia** | **Hyperglycemia** | -.4693^*^ | .10135 | .000 | -.6679 | -.2706 |
|  |  | **Borderline hyperglycemia** | -.0383 | .10633 | .718 | -.2468 | .1701 |
| **RBC** | **Hyperglycemia** | **Borderline hyperglycemia** | .0424^*^ | .01776 | .017 | .0076 | .0772 |
|  |  | **Normoglycemia** | .0859^*^ | .01678 | .000 | .0530 | .1188 |
|  | **Borderline hyperglycemia** | **Hyperglycemia** | -.0424^*^ | .01776 | .017 | -.0772 | -.0076 |
|  |  | **Normoglycemia** | .0435^*^ | .01761 | .013 | .0090 | .0781 |
|  | **Normoglycemia**  **Hyperglycemia** | **Hyperglycemia** | -.0859^*^ | .01678 | .000 | -.1188 | -.0530 |
|  |  | **Borderline hyperglycemia** | -.0435^*^ | .01761 | .013 | -.0781 | -.0090 |
| **MCH** | **Hyperglycemia** | **Borderline hyperglycemia** | -.202^*^ | .0802 | .012 | -.359 | -.045 |
|  |  | **Normoglycemia** | -.211^*^ | .0758 | .005 | -.360 | -.063 |
|  | **Borderline hyperglycemia** | **Hyperglycemia** | .202^*^ | .0802 | .012 | .045 | .359 |
|  |  | **Normoglycemia** | -.009 | .0796 | .908 | -.165 | .147 |
|  | **Normoglycemia**  **Hyperglycemia** | **Hyperglycemia** | .211^*^ | .0758 | .005 | .063 | .360 |
|  |  | **Borderline hyperglycemia** | .009 | .0796 | .908 | -.147 | .165 |
| **MCHC** | **Hyperglycemia** | **Borderline hyperglycemia** | .009 | .0381 | .809 | -.065 | .084 |
|  |  | **Normoglycemia** | -.022 | .0360 | .534 | -.093 | .048 |
|  | **Borderline hyperglycemia** | **Hyperglycemia** | -.009 | .0381 | .809 | -.084 | .065 |
|  |  | **Normoglycemia** | -.032 | .0378 | .403 | -.106 | .042 |
|  | **Normoglycemia**  **Hyperglycemia** | **Hyperglycemia** | .022 | .0360 | .534 | -.048 | .093 |
|  |  | **Borderline hyperglycemia** | .032 | .0378 | .403 | -.042 | .106 |
| **MCV** | **Hyperglycemia** | **Borderline hyperglycemia** | -.658^*^ | .2039 | .001 | -1.057 | -.258 |
|  |  | **Normoglycemia** | -.568^*^ | .1927 | .003 | -.945 | -.190 |
|  | **Borderline hyperglycemia** | **Hyperglycemia** | .658^*^ | .2039 | .001 | .258 | 1.057 |
|  |  | **Normoglycemia** | .090 | .2022 | .657 | -.306 | .486 |
|  | **Normoglycemia** | **Hyperglycemia** | .568^*^ | .1927 | .003 | .190 | .945 |
|  |  | **Borderline hyperglycemia** | -.090 | .2022 | .657 | -.486 | .306 |
| **HCT** | **Hyperglycemia** | **Borderline hyperglycemia** | .044 | .1456 | .762 | -.241 | .329 |
|  |  | **Normoglycemia** | .422^*^ | .1376 | .002 | .153 | .692 |
|  | **Borderline hyperglycemia** | **Hyperglycemia** | -.044 | .1456 | .762 | -.329 | .241 |
|  |  | **Normoglycemia** | .378^*^ | .1443 | .009 | .095 | .661 |
|  | **Normoglycemia** | **Hyperglycemia** | -.422^*^ | .1376 | .002 | -.692 | -.153 |
|  |  | **Borderline hyperglycemia** | -.378^*^ | .1443 | .009 | -.661 | -.095 |
| **Hb** | **Hyperglycemia** | **Borderline hyperglycemia** | .018 | .0520 | .729 | -.084 | .120 |
|  |  | **Normoglycemia** | .133^*^ | .0491 | .007 | .036 | .229 |
|  | **Borderline hyperglycemia** | **Hyperglycemia** | -.018 | .0520 | .729 | -.120 | .084 |
|  |  | **Normoglycemia** | .115^*^ | .0516 | .026 | .014 | .216 |
|  | **Normoglycemia** | **Hyperglycemia** | -.133^*^ | .0491 | .007 | -.229 | -.036 |
|  |  | **Borderline hyperglycemia** | -.115^*^ | .0516 | .026 | -.216 | -.014 |
| **FBG** | **Hyperglycemia** | **Borderline hyperglycemia** | 66.276^*^ | .9767 | .000 | 64.362 | 68.191 |
|  |  | **Normoglycemia** | 89.322^*^ | .9231 | .000 | 87.512 | 91.131 |
|  | **Borderline hyperglycemia** | **Hyperglycemia** | -66.276^*^ | .9767 | .000 | -68.191 | -64.362 |
|  |  | **Normoglycemia** | 23.046^*^ | .9684 | .000 | 21.147 | 24.944 |
|  | **Normoglycemia** | **Hyperglycemia** | -89.322^*^ | .9231 | .000 | -91.131 | -87.512 |
|  |  | **Borderline hyperglycemia** | -23.046^*^ | .9684 | .000 | -24.944 | -21.147 |
| Based on observed means.  The error term is Mean Square (Error) = 1265.336.  * The mean difference is significant at the .05 level. | | | | | | | |
